# Induced Fit in Protein Multimerization: The HFBI Case

**DOI:** 10.1371/journal.pcbi.1005202

**Published:** 2016-11-10

**Authors:** Laura Riccardi, Paolo Mereghetti

**Affiliations:** 1 Laboratory of Molecular Modeling and Drug Discovery, Istituto Italiano di Tecnologia, Genoa, Italy; 2 Center for Nanotechnology Innovation @NEST, Istituto Italiano di Tecnologia, Pisa, Italy; Koç University, TURKEY

## Abstract

Hydrophobins, produced by filamentous fungi, are small amphipathic proteins whose biological functions rely on their unique surface-activity properties. Understanding the mechanistic details of the multimerization process is of primary importance to clarify the interfacial activity of hydrophobins. We used free energy calculations to study the role of a flexible *β*-hairpin in the multimerization process in hydrophobin II from *Trichoderma reesei* (HFBI). We characterized how the displacement of this *β*-hairpin controls the stability of the monomers/dimers/tetramers in solution. The regulation of the oligomerization equilibrium of HFBI will necessarily affect its interfacial properties, fundamental for its biological function and for technological applications. Moreover, we propose possible routes for the multimerization process of HFBI in solution. This is the first case where a mechanism by which a flexible loop flanking a rigid patch controls the protein-protein binding equilibrium, already known for proteins with charged binding hot-spots, is described within a hydrophobic patch.

## Author Summary

Fungi proliferate by creating a complex hyphal network growing within a wet environment. However, for most fungi to colonize new territories, they must produce spores carried by aerial hyphae and spread them into the air. Aerial structures need to overcome the surface tension of the surrounding water in order to grow into the air. This process requires hydrophobins, a remarkable class of self-associating fungal proteins which lower the surface tension at the air/water interface by creating a thin amphipathic layer. In solution they form multimers in equilibrium with the interfacial layer. Due to their unique surface-activity properties, hydrophobins have been used for a variety of biotechnical applications. We used enhanced sampling molecular dynamics simulations methods to study the multimerization process in solution of a hydrophobin from *Trichoderma reesei* (HFBI). We clarified the fundamental role of a small flexible region within the HFBI monomer involved in the formation of multimers. A flexible loop flanking a rigid interaction patch is able to fine-tune the interaction energy. This mechanism, already known for charged binding patches, is described here for hydrophobic hot-spots. This result is remarkably important in order to clarify the mechanism of arranging at the interface and enhancing hydrophobin-based technological applications.

## Introduction

Hydrophobins are small (7–15 kDa) proteins produced by filamentous fungi. They are globular and rigid proteins containing four disulfide bridges which stabilize the structure. Hydrophobins perform a variety of biological roles at interfaces that help fungi to adapt to their environment including adhesion and coatings of spores. Moreover, hydrophobins lower the surface tension of water so that fungal hyphae can penetrate the air-water interface and grow outside aqueous media [1–3]. The remarkable surface-activity properties of hydrophobins come from their amphiphilic nature. Besides their amphiphilicity, specific intermolecular interactions also contribute to their functional properties [4–9]. Due to their unique properties, hydrophobins have become attractive for use in several types of biotechnical applications. These include stabilization of colloidal dispersions, reverse the wettability of surfaces, dispersion of insoluble drug compounds, production of stable foams, and protein immobilization [8, 10–13]. Hydrophobins are very soluble in water up to 100 mg/mL and display unusual detergent-like behaviour in solution as they form different kinds of oligomers, depending on the conditions and on the hydrophobin type [9, 14, 15].

Hydrophobins have been divided into two classes, class I and class II, based on the hydropathy profile of the amino-acid sequence [16]. In particular, class I hydrophobins are more resistant to dissociation using solvents and detergents than class II hydrophobins. Furthermore, class I hydrophobins tend to form rodlet-like aggregates at interfaces, whereas for class II hydrophobins various needle-like crystals and structured surface films have been observed [17–19]. The work described here was done on HFBI, a class II hydrophobins of the fungus *Trichoderma reesei*.

The crystal structure of HFBI from *T. reesei*, solved in 2006 by Hakanpää and colleagues (PDB id: 2FZ6), shows four molecules in the asymmetric unit [20]. A tetrameric structure was also found in solution, where HFBI forms oligomers (dimers and tetramers) in a concentration-dependent manner. In solution, the tetramer is slightly larger and more elongated, with its monomers not as tightly packed as in the crystal. The oligomers are in some ways analogous to micelles, however, with the clear difference that the HFBI oligomers contain only two or four molecules [4]. Above a critical concentration (20 *μ*M), HFBI is mainly in tetrameric form [9]. Besides oligomers, HFBI shows strong surface activity. HFBI is indeed a protein that self-organizes to form precise membrane structures [4, 18, 19, 21, 22]. Hydrophobin multimerization was suggested to protect the hydrophobic parts and that these associations disassemble at the interface to form monolayers. At the interface, HFBI exists as monomers, oligomers and surface monolayers, and the equilibrium is shifted towards surface assemblies [9, 20].

Powers and colleagues [23] have shown that the mechanism of protein tetramerization via dimers is evolutionally favored over tetramerization via monomers and trimers. It is likely that the multimerization process of HFBI involves combination of monomers to dimers with the successive combination of dimers to tetramers [9, 14].

**Fig 1:**
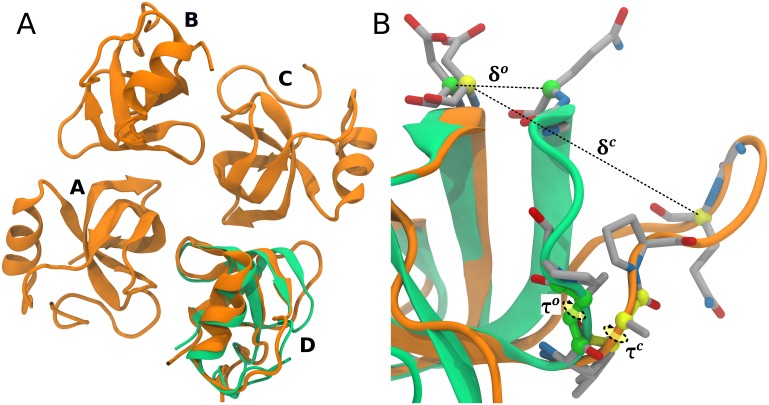
HFBI 3D structure and collective variables. (A) In orange, crystal structure of the HFBI protein (PDB id: 2FZ6). On chain D (*o* conformation), the structure of chain A (*c* conformation) is superposed in green. (B) Details of the region enclosing loop 60–66 showing the collective variables (*δ* and *τ*) used in the metadynamics simulations. Conformations *c* (green) and *o* (orange) are superposed.

In the HFBI structure, there are two types of molecules with respect to the conformation of the second *β*-hairpin motif (residues 60 to 66). Molecules A and C had this area in a similar “closed” conformation while molecules B and D both possessed an “open” conformation. The central *β*-barrel structure, with four disulfide bridges, remains unchanged [20], see Fig 1. In this paper, “closed” conformation of monomeric units A and C is named *c*, while “open” conformation of molecules B and D, is called *o*. It was suggested that movement in the *β*-hairpin area was most likely driven by the formation of the HFBI tetramer [20].

In a recent computational study, it was found that dimers and tetramers encounter complexes only form when monomers are in *c* conformation [24]. This supports the idea of an induced conformational transition upon encounter complex formation. In this work we explored the multimerization process of HFBI in solution. The fundamental role of the last *β*-hairpin in the oligomeric assembly is unveiled using all-atoms metadynamics simulations and a plausible oligomerization pathway is proposed.

## Materials and Methods

### Structural models

All the computational models of HBFI here considered are based on the X-ray structure from *Trichoderma reesei*, solved at 2.1 Å resolution (PDB id: 2FZ6) [20]. This structure is an hetero-tetramer with each unit consisting of 75 residues. The monomers are characterized by a different position of the second *β*-hairpin (residues 60 to 66) with respect to the central *β*-barrel. In particular, chains B and D are in the so called conformation *o*, with the second *β*-hairpin exposed to the solvent, while chains A and C are in conformation *c*, with the second *β*-hairpin closed to the protein core. In the models, the starting units correspond to a specific chain in the crystal. Monomer(*c*) is chain A; monomer(*o*) is chain D; dimer(*cc*) is chain C + chain A (superposed on chain D of crystal); tetramer(*cccc*) is chain A + chain A (superposed on B-C-D); tetramer(*cocc*) is chain A + chain B + chain A (superposed on C-D); and tetramer(*coco*) is the crystal structure, see S2 Fig. The chain subjected to metadynamic bias is given in bold typeface (see section “Well-Tempered Metadynamics (MetaD)”).

### Standard molecular dynamics (MD) simulations

For each system (monomer/dimer/tetramer), we followed the simulation protocol described hereinafter. The protein was put in a dodecahedric box of TIP3P water molecules ensuring a minimum distance to the box edges of 1 nm. The monomeric systems are neutral, while the dimer and tetramer have positive charge due to the presence of the Zn^2+^ ions at the interface between chains A/B and between chains C/D. The proper amount of Na^+^ and Cl^−^ ions was added to reach a ionic concentration of 150 mM and ensure final neutral systems (see S1 Table). A steepest-descent minimization was applied to relax the solvent molecules around the solute. The equilibration was performed in two steps: the system was at first thermalized up to 300 K coupling the protein and the solvent to a V-rescale thermostat [25] (*τ*_*t*_ = 0.1) in the canonical ensemble (NVT). Then, we switched to the NPT statistical ensemble, performing 100 ps of MD at 300 K, coupling the system with a Parrinello-Rahman barostat [26] (*τ*_*p*_ = 2). After this initial phase the system was ready for productive MD simulations. Production runs were carried out in the NPT (p = 1 bar, T = 300 K) statistical ensemble. All bonds were constrained with LINCS [27], allowing to use a time step set of 2 fs. Periodic boundary conditions were applied to the systems in all directions. PME method [28] was used to evaluate long-range electrostatic interactions (pme order = 4, fourier spacing = 0.12), and a cutoff of 10 Å was used to account for the van der Waals interactions. Coordinates of the systems were collected every 2 ps. All MD simulations were carried out with GROMACS-5 [29] using the AMBER99 force field [30] on GPU/CPU machines. The length of the MD simulations was of 150 ns, for monomer(*c*) and monomer(*o*), 100 ns, for dimer(*cc*), and 300 ns, for tetramer(*cccc*) and tetramer(*coco*) (see S1 Table). Within each monomeric unit four covalent crosslinks between CYS^18^-CYS^48^, CYS^19^-CYS^31^, CYS^8^-CYS^57^, and CYS^58^-CYS^69^ (see S1 Fig) have been defined and treated according to the disulfide bridge parameterization as in AMBER99 force field [30]. Standard MD were used for guessing CVs for metadynamics simulations [31] and for all analysis other then the free energy calculations.

### Well-tempered metadynamics (MetaD)

Well-tempered metadynamics, labelled as MetaD, simulations were performed with GROMACS-5 [29] using AMBER99 force field [30] and the PLUMED version 2.2 [32] plugin for free energy calculations. The starting structure for MetaD were taken after the NPT equilibration described above. The collective variables (CVs) used to describe the transition between monomer(***c***) and monomer(*o*) were the distance $\delta =[{\text{ASP}}_{\mathrm{CA}}^{30}-{\text{GLN}}_{\mathrm{CA}}^{65}]$, and the torsion $\tau =[{\text{VAL}}_{C}^{59}-{\text{ALA}}_{N}^{60}-{\text{ALA}}_{CA}^{60}-{\text{ALA}}_{C}^{60}]$. Both variables were necessary to properly describe the transition *c*/*o* without irreversibly distorting the structure of the *β*-hairpin. Metadynamics bias was constructed adding a Gaussian function with an initial height of 1.2*T/*T*_0_ kJ/mol and a width of 0.1. T_0_ was set to 300 K and the bias factor (*γ* = (*T* + Δ*T*)/*T*) was set to 10. An upper wall at 1.3 nm with a *κ* of 2000 kJ/mol/nm^2^ was associated to the CV *δ*. This choice was justified by the fact that in the open conformation *o*, the value of *δ* is 1.2 nm. In multimers the metadynamic bias was applied only to chain D, highlighted in bold typeface when specified in the text. For example, performing MetaD on tetramer(*ccc**c***) means that the starting structure was a tetramer composed of four *c* conformations and the MetaD bias was applied to chain D.

**Fig 2:**
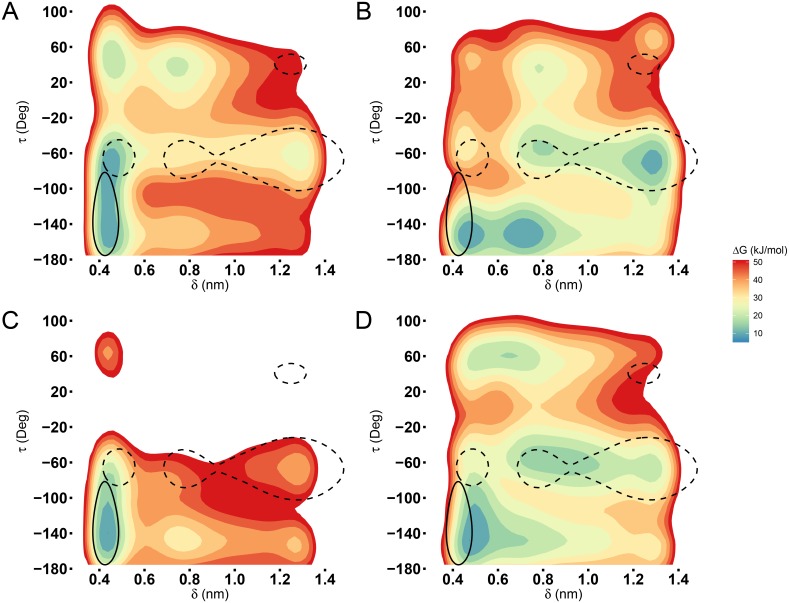
Free energy landscapes. Free energy landscapes obtained from MetaD simulations on the collective variables *δ* and *τ* for (A) monomer(***c***), (B) dimer(*c****c***), (C) tetramer(*ccc****c***), and (D) tetramer(*coc****c***). Superposed contour lines define regions which enclose 90% of the conformations sampled by the *c* (continuous) and *o* (dashed) form during 300 ns standard MD simulations.

Convergence was checked by computing the free energy as a function of simulation time (10 ns blocks). Moreover, the value of FEP(*δ*) at $\delta_{{min}_{3}}\;=\;1.25\text{ nm}$ nm has been plotted as function of time. At convergence, the reconstructed profiles should be similar, and the value at $\delta_{{min}_{3}}\;=\;1.25\text{ nm}$ should be constant (see S3 Fig). Each MetaD simulation is 200 ns long, enough to ensure a proper convergence of the free-energy (all details in the SI). In order to obtain reference regions on the CVs space sampled by the *c* and the *o* forms, the joint probability density function *f*(*δ*, *τ*) has been computed from standard MD simulations of monomer(*c*) and tetramer(*coco*). In the tetramer case, *f*(*δ*, *τ*) has been computed as an average across the two monomeric units in *o* form. Contour levels specifying the *c* and *o* regions on the FES plots (black lines in Fig 2) are specified as volume percentages.

For example, a contour at 90% encloses the 90% of the most probable data points and excludes the remaining 10%. The contour volume percentage can be specified as follow: given a joint probability density function *f*_*i*_(*δ*, *τ*) we want to find the set *A* which includes all points *i* such that

$$\sum_{i\in A}f_{i}\left(\delta ,\tau \right)d\delta d\tau =\chi$$

where *χ* is, for example, 0.9. In order to compute the contour volume percentage the following algorithm is used: i) sort all points i according to the value of *f*_*i*_(*δ*, *τ*) in decreasing order obtaining the ordered list $L={\left\{i_{k}\right\}}_{k=1}^{k=N}$. ii) Compute the cumulative sum on the sorted values, *C* = *cumsum*(*L*). iii) Compute *Z* = ∑_*i*_ *f*_*i*_(*δ*, *τ*). iv) Set *A* is defined by all *i*_*k*_ such that *C* ≤ 0.9*Z*. The isocontour line is defined by all *i*_*k*_ such that *C* ≡ 0.9*Z*.

### Free energy surfaces (FES)

Two-dimensional free energy surfaces as a function of *δ* and *τ* have been obtained by summation of the added Gaussian hills. The 2D surface was discretized using a spacing of 0.035 nm and 0.035 deg on *δ* and *τ*, respectively.

### Free energy profiles (FEP)

**Fig 3:**
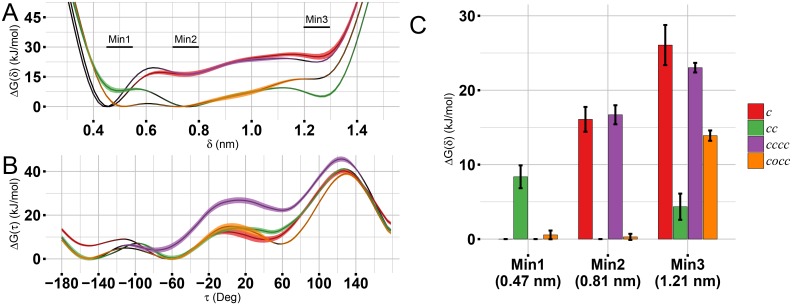
Free energy profiles. Superposed free energy profiles calculated from MetaD simulations using (A) *δ* and (B) *τ* collective variable. Shadows around each line represent the standard error of the mean, n = 5 (simulations blocks). On panel C values of ΔG located at the three main minima on the free energy profile over the collective variable *δ* are compared. The height of the bar shows the mean value of 5 simulations blocks. Error bars represent the 95% confidence interval.

Free energy profiles as a function of a single CV, FEP(*δ*) and FEP(*τ*), have been computed integrating out one CV from the two-dimensional FES(*δ*, *τ*) (Fig 3A and 3B). The FEP(*δ*) as a function of simulation time (every 10 ns blocks) was computed and the last 5 blocks were used to estimate the mean $\langle FEP\left(\delta \right)\rangle =\frac{1}{N}\sum_{i}^{5}FEP_{i}\left(\delta \right)$ and the standard error of the mean as $se_{FEP(\delta )}=\frac{\sigma_{FEP(\delta )}}{\sqrt{n}}$, where *σ*_*FEP*(*δ*)_ is the standard deviation across the five simulation blocks (*n* = 5). Throughout the paper, the angular brackets for the average FEP were dropped for clarity. A similar procedure was applied for the other CV, *τ*. On FEP(*δ*), three free energy minima have been selected as representative of *c* conformation ($\delta_{{min}_{1}}\;=\;0.47\text{ nm}$) and *o* conformation ($\delta_{{min}_{2}}\;=\;0.81\text{ nm}$ and $\delta_{{min}_{3}}\;=\;1.25\text{ nm}$). The free energy values at the three minima have been computed for monomer(***c***), dimer(*c**c***), tetramer(*ccc**c***), and tetramer(*coc**c***) and plotted as mean ± the 95% confidence interval, $CI^{95\%}\;=\;\Delta G(\delta_{{min}_{i}})\;\pm \;1.96se$.

### Hydrogen bonds

**Fig 4:**
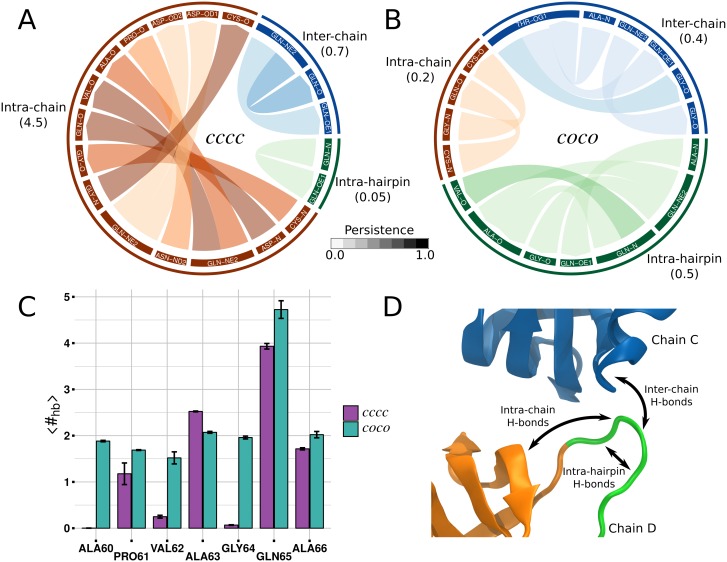
Hydrogen bonds network analysis. Chord diagram showing the H-bonds formed by the aminoacids in the *β*-hairpin and the rest of the molecule in (A) tetramer(*cccc*) and (B) tetramer(*coco*). Directionality of H-bonds (donor→acceptor) is represented with an arrow in the connection link. Links transparency indicates the H-bonds persistence, lighter color lower persistence, a grayscale colorbar is given as a guide for the eyes. In parentheses, the average number of H-bonds per frame is given. (C) Average number of H-bonds per frame formed between the *β*-hairpin and solvent, for each residue. H-bonds decomposition into intra-hairpin, intra-chain and inter-chain groups is schematically described on panel D. H-bonds analysis was performed on standard MD simulations.

Hydrogen bonds were calculated using GROMACS-5 software tools on the 300 ns standard MD simulations for tetramer(*cccc*) and tetramer(*coco*). The H-bond persistence was computed as the number of times the i^*th*^ H-bond was found, divided by the total number of frames. Only H-bonds with persistence > 5% were retained for the analysis. We decomposed the hydrogen bonds into four groups: i) intra-hairpin, ii) intra-chain, iii) inter-chain, and iv) hairpin-solvent. The intra-hairpin includes hydrogen bonds formed within the residues 60–66 of the *β*-hairpin. The intra-chain group corresponds to the hydrogen-bonds between the *β*-hairpin and the rest of the chain. The inter-chain group contains hydrogen-bonds established between the *β*-hairpin and the chain facing the *β*-hairpin. Finally, hairpin-solvent group includes hydrogen bonds formed by the residues of the *β*-hairpin and the solvent (see Fig 4D for a description of the groups). While for groups i, ii and iii an atomistic detail was considered, for group iv, the average number of hydrogen bonds formed between a given aminoacid and the water was used. H-bonds analysis was performed on one monomeric unit (chain D) within tetramer(*cccc*) as well as tetramer(*coco*).

### Solvation free energy

Solvation free energy has been computed using software *g*_*mmpbsa*_ [33]. Briefly, the solvation free energy is expressed as sum of two terms *G*_*solvation*_ = *G*_*polar*_ + *G*_*non* − *polar*_. *G*_*polar*_ is obtained solving the linearized Poisson-Boltzmann equation using APBS software [34]. Ionic strength was set to 150 mM, solute and solvent static dielectric constants were set to 2.0 and 78.4 respectively. *G*_*non* − *polar*_ was computed using the solvent accessible surface area (*SASA*) model [33] as *G*_*non* − *polar*_ = *γSASA* + *b*, where *γ* is a coefficient related to surface tension of the solvent and was set to 0.0226778 kJ mol^−1^ Å^−2^, and b = 3.84982 kJ/mol is a fitting parameter. Hundred equally spaced frames were extracted from standard NPT molecular dynamics simulations of monomer(*c*), monomer(*o*), dimer(*cc*), dimer(*co*), tetramer(*cccc*), and tetramer(*coco*). Frames were separated by at least 1 ns (depending on total simulation length, see S1 Table for the simulations details) from each other in order to avoid correlations. Δ*G*_*polar*_ and Δ*G*_*non* − *polar*_ terms were computed on each frame. Statistical analysis was performed comparing pairs monomer(*c*)/monomer(*o*), dimer(*cc*)/dimer(*co*), and tetramer(*cccc*)/tetramer(*coco*) using a Welch’s t-test. p<0.01 was considered statistically significant.

### Electrostatic potential

**Fig 5:**
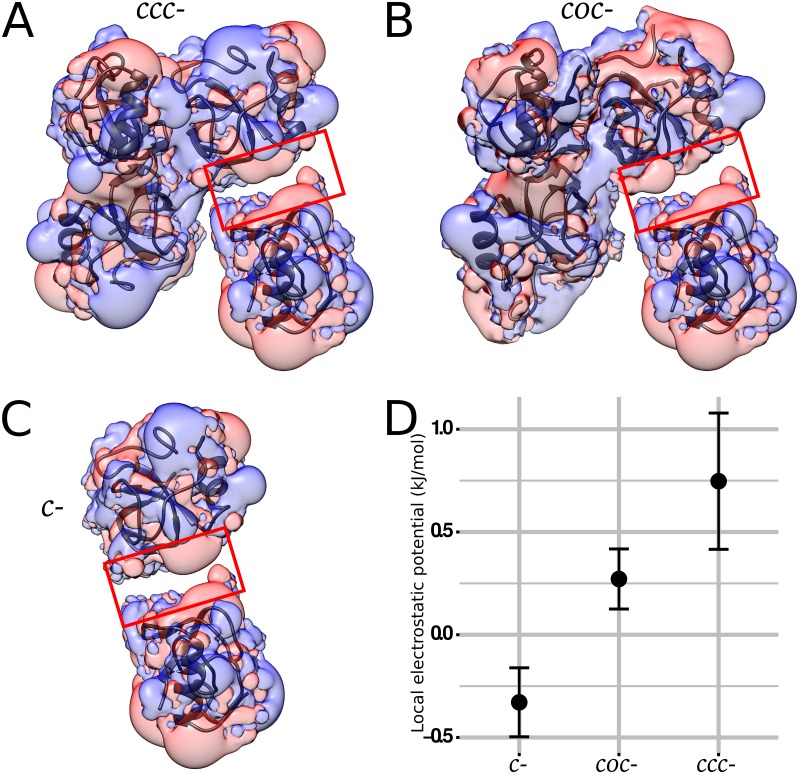
Electrostatic potential. (A, B) Electrostatic potential computed for chains A-B-C and chain D (separately) for tetramer(*cccc*) and tetramer(*coco*), respectively. (C) electrostatic potential computed for chain C and chain D (separately) in dimer(*c****c***). As a reference structure the centroid of the most populated cluster obtained from a cluster analysis performed on standard MD simulations was used. Iso-surfaces were taken at ±1 kJ/mol. In the picture, chain D was manually displaced to avoid overlap with the other chains and having a clearer view. Negative potential is shown in red while positive potential in blue. (D) Mean ± s.e.m. electrostatic potential at the interface between chain C and D, see Methods for the calculation details. The red cuboid shows the subregion used to compute the electrostatic potential at the interface shown in panel (D). The codes *ccc*−, *coc*−, and *c*− remark that the electrostatics is calculated taking chain D apart.

The electrostatic potential was obtained solving the linearized Poisson-Boltzmann equation using APBS software [34]. Ionic strength was set to 150 mM, solute and solvent static dielectric constants were set to 2.0 and 78.4 respectively. The single sphere Debye-Hükel model was used as boundary condition for coarse grid. Smoothed molecular surface was used to define the dielectric boundaries. The electrostatic potential has been computed separately for chains A-B-C and chain D in tetramer(*cccc*) and tetramer(*cocc*), chain C and chain D, separately, in dimer(*cc*). A cluster analysis was performed on standard MD simulations (see S1 Table for details about simulations parameters) using single linkage algorithm setting 0.15 nm as RMSD cutoff. The centroid of the most populated cluster was used as reference structure for the calculation of the electrostatic potential maps in Fig 5A, 5B and 5C. In order to estimate a local electrostatic potential at the interface between chains C and D, the potential was averaged within a cuboid subregion enclosing the C/D interface. The subregion was defined as normal to the plane formed by the *β*-sheet of chain D at the C/D interface and with sides of length 2.0, 2.0, and 1.0 nm, see Fig 5A, 5B and 5C. This local electrostatic potential was computed over the entire standard MD trajectory using conformations every 1 ns. Mean value and standard error of the mean (s.e.m) have been then obtained, see Fig 5D.

### Interface analysis

Interfaces between all monomeric units of tetramer(*cccc*), tetramer(*cocc*), and tetramer(*coco*), have been computed from the entire standard MD simulations. The following chain pairs have been considered: A/B, B/C, C/D, A/D, B/D, and A/C. Each interface has been described in terms of interface area, distance maps, and residues at the interface. For the pairs of chains *i*/*j* the interface area has been computed as *SASA*_*i*/*j*_ = (*SASA*_*i*_ + *SASA*_*j*_) − *SASA*_*i*,*j*_, where *SASA*_*i*,*j*_ is the *SASA* computed for the complex *i*/*j*, while *SASA*_*i*_ and *SASA*_*j*_ are the *SASA* of the isolated chains. Solvent accessible surface area was computed using the GROMACS tool *sasa* [29]. Distance maps have been obtained by measuring the smallest distances between residue pairs (heavy atoms only) for all trajectory frames and averaging over time. Interacting residues have been defined as pairs of aminoacids whose distance was up to 0.45 nm on the distance map [35]. The GROMACS tool *mdmat* [29] was used for this purpose.

### Data analysis and figures

All data and statistical analysis were performed using the software package R version 3.2 [36]. Figures for the three-dimensional protein structures have been obtained using VMD version 1.9.2 [37] and Chimera version 1.10 [38].

## Results

### Free energy surface of *β*-hairpin rearrangement

The role of the last *β*-hairpin in the oligomeric assembly was probed by exploring the transition from conformation *c* to *o* using metadynamics (MetaD) [31]. The MetaD bias was applied to two configurational collective variables (CVs): the distance $\delta =[{\text{ASP}}_{\mathrm{CA}}^{30}-{\text{GLN}}_{\mathrm{CA}}^{65}]$, and the torsion $\tau =[{\text{VAL}}_{C}^{59}-{\text{ALA}}_{N}^{60}-{\text{ALA}}_{CA}^{60}-{\text{ALA}}_{C}^{60}]$ (Fig 1). These CVs where empirically selected observing the *β*-hairpin motion, in standard MD simulations, of the monomer in open and closed forms. While the distance *δ* is clearly an obvious coordinate for describing the opening of the *β*-hairpin, torsion *τ* has been selected as this dihedral angle changes from ≈-150 deg to ≈-60 deg from the closed to the open conformation (Fig 1). As mentioned in method section, upper/lower bounds were added to these CVs to avoid the unfolding of the protein structure. In order to understand the influence of the multimerization process on the conformational rearrangement, four MetaD simulations have been performed starting from monomer(***c***), dimer(*c**c***), tetramer(*ccc**c***), and tetramer(*coc**c***) conformations. Convergence of the MetaD simulations has been assessed as described in the Method Section. The free energy surfaces as a function of *δ* and *τ*, FES(*δ*, *τ*), are reported in Fig 2 (see also the corresponding probability density functions in S4 Fig). FES have been shifted as *min*(FES(*δ*, *τ*)) = 0. At 300 K, the *β*-hairpin is flexible so, standard MD simulations have been performed for the monomer in conformation *c* (monomer(***c***)) and for the tetrameric crystal structure (tetramer(*coco*)) to obtain reference regions on the CVs space sampled by the *c* and the *o* forms. From these MD simulations, percentage volume contours enclosing 90% of the most probable conformations were plotted over the FES(*δ*, *τ*) in order to locate the *c* (continuous line) and *o* (dashed line) conformation. Considering the MetaD simulations of the monomer, a main minimum was found at $\delta_{{min}_{1}}\;=\;0.47\text{ nm}$, *τ* = [-150, -60] deg which corresponds to the *c* form (Fig 2A). In solution the equilibrium distribution of the HFBI monomer is shifted to the *c* conformation. The dimer shows a different behaviour, three main minima appears on the surface, at $\delta_{{min}_{1}}\;=\;0.47\text{ nm}$, *τ* = -150 deg; $\delta_{{min}_{2}}\;=\;0.81\text{ nm}$, *τ* = -150 deg; and $\delta_{{min}_{3}}\;=\;1.25\text{ nm}$, *τ* = -60 deg. A video showing the MetaD simulation of the dimer can be found in SI, S1 Video.

The FES(*δ*, *τ*) of the homo-tetramer *cccc* is similar to the FES of the monomer in solution, i.e. the thermodynamically favoured state is the *c* conformation.

MetaD simulations were performed starting from the hetero-tetramer *cocc* in order to assess for a cooperative effect in the conformational rearrangement (*c* to *o* state) of one monomeric unit depending on the presence of a second monomer in the *o* form. The FES(*δ*, *τ*) of hetero-tetramer resembles the one of the dimer, where multiple main minima exist. In particular, two broad minima are visible around $\delta_{{min}_{1}}\;=\;0.47\text{ nm}$, *τ* = -150 deg, and $\delta_{{min}_{2}}\;=\;0.81\text{ nm}$, *τ* = -60 deg.

To summarize the differences between the four MetaD cases, free energy profiles as a function of a single CV, FEP(*δ*), FEP(*τ*) have been computed integrating out one CV from the two-dimensional FES(*δ*, *τ*) (Fig 3A and 3B). From there, it is clear the different behaviour of the monomer(***c***) and the tetramer(*ccc**c***) compared to the dimer(*c**c***) or the tetramer(*coc**c***) forms. To quantify those differences and assess their statistical significance, the values of the main free energy minima on the most representative collective variable, the distance *δ*, have been compared, Fig 3C. Considering the distance as unique CV, the *c* conformation is identified by $\delta_{{min}_{1}}\;=\;0.47\text{ nm}$, while the *o* conformation is defined by $\delta_{{min}_{2}}\;=\;0.81\text{ nm}$ and $\delta_{{min}_{3}}\;=\;1.25\text{ nm}$. Monomer(***c***) and tetramer(*ccc**c***) have a pronounced minimum at $\delta_{{min}_{1}}\;=\;0.47\text{ nm}$ while the other two distances ($\delta_{{min}_{2}}\;=\;0.81\text{ nm}$,$\delta_{{min}_{3}}\;=\;1.25\text{ nm}$) have large free energy values. Conversely, in the dimer(*c****c***) the equilibrium distance is shifted toward $\delta_{{min}_{2}}\;=\;0.81\text{ nm}$ and $\delta_{{min}_{3}}\;=\;1.25\text{ nm}$, i.e. the *o* form. In tetramer(*coc****c***), the profile is flatter with nearly zero free energy value for $\delta_{{min}_{1}}\;=\;0.47\text{ nm}$ and $\delta_{{min}_{2}}\;=\;0.81\text{ nm}$ which confirm an intermediate behaviour between dimer(*c**c***) and tetramer(*ccc**c***). In MetaD simulations, the monomeric units not subjected to MetaD bias remain in their initial configurational state (*c* or *o*). This has been checked by plotting the values of *δ* and *τ* for chains A, B and C in the tetramers and chain C in the dimer for all MetaD simulations, see S5 Fig.

### Role of the hydrogen bonds in the *β*-hairpin rearrangement

Hydrogen bonds (H-bonds) formed by the aminoacids in the *β*-hairpin and the rest of the molecule affect the stability of the *c*/*o* conformations and can explain why the *β*-hairpin opens within dimer(*c**c***) or tetramer(*coc**c***) and remains closed in monomer(***c***) or tetramer(*ccc**c***). We decomposed the H-bonds into four groups: i) intra-hairpin, ii) intra-chain and iii) inter-chain, and iv) hairpin-solvent, see Methods Section for details. In Fig 4A and 4B the H-bonds and their average persistence is shown in a chord diagram for group i, ii and iii, see also S2 Table for quantitative information about the H-bonds networks. Considering tetramer(*cccc*), several persistent H-bonds are present between the *β*-hairpin and the rest of the chain, which is expected as the *β*-hairpin is parallel to a *β*-strand. Almost no H-bond is found within the *β*-hairpin itself. Two slightly persistent H-bonds form between the *β*-hairpin and the facing chain (Fig 4A). Focusing on tetramer(*coco*), it is clear that the drastic reduction of intra-chain H-bonds is due to the *β*-hairpin opening. Despite the persistence is low, several H-bonds form within the *β*-hairpin itself and some with the interfacing chain (Fig 4B). In tetramer(*coco*), the H-bonds persistence is lower and the average number of H-bonds is also smaller compared to tetramer(*cccc*). However, looking at the average number of H-bonds formed by the residues within the hairpin and the solvent, the picture is inverted (Fig 4C). The *β*-hairpin opening exposes its mainchain to the solvent allowing the formation of stable H-bonds with water molecules. In particular, approximately two H-bonds are gained for ALA60, VAL62 and GLY64 in the transition from *c* to *o* conformation. Recalling that the stable conformation of the monomer in solution is the *c* form, the opening of the *β*-hairpin in dimer(*c**c***) can not only depend on solvent mediated H-interactions, i.e. a large hydrophobic patch is present on the surface of the HFBI monomer and may affect its stability and the *β*-hairpin rearrangement.

### Energetic contributions in *o*/*c* transition

**Fig 6:**
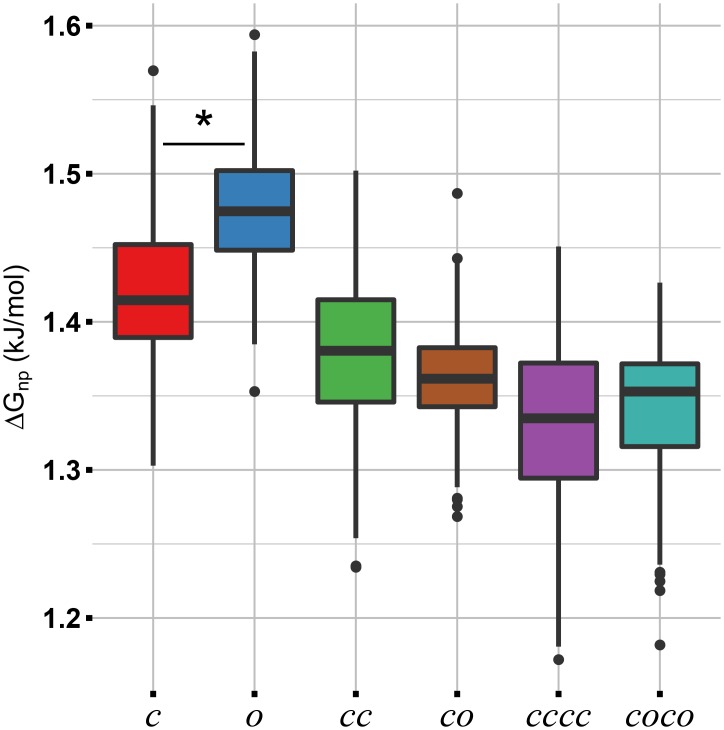
Non-polar solvation free energy. Non-polar contribution to the solvation free energy computed for different oligomerization states. The Welch’s t-test revealed statistically significant differences between pairs *c*/*o* (*t*_196.5_ = -8.9, p<0.001). In a boxplot, the box contains 50% of the distribution (from the first to the third quartile) and the whiskers extend to the most extreme values of the distribution (that is, 1.5 times the width of the box). Black dots represent outliers; n = 100. Analysis was performed on standard MD simulations.

Polar (Δ*G*_*polar*_) and non-polar (Δ*G*_*non*−*polar*_) contribution to the solvation free energy have been calculated using APBS [34] on 100 structures extracted from equilibrium simulations. Non-polar contribution has been computed using the solvent accessible surface area model [33] (further details in the Method Section). To assess for differences between *c* and *o* forms in different oligomerization states, Δ*G*_*non*−*polar*_ was calculated for the following pairs: monomer(*c*)/monomer(*o*), dimer(*cc*)/dimer(*co*), and tetramer(*cccc*)/tetramer(*coco*) (Fig 6). Pair monomer(*c*)/monomer(*o*) shows statistically significant (Welch’s t-test, t = -8.9, df = 196.5, p<0.001) difference in Δ*G*_*non*−*polar*_, with the *o* conformation having a large free energy value.

Close to the *β*-hairpin, e.g. at the interface between chain C and chain D, the electrostatic potential varies depending on whether conformation of chain B is *c* or *o* and if chain B is present or not, see Fig 5 and S2 Video. In the very same region, the electrostatic potential of chain D in *c* form is also negative, creating an electrostatic clash between chains D and chain C. The local electrostatic potential at the C/D interface is negative in *c*−, and positive in *coc*− and *ccc*−, see Fig 5D. The presence of an electrostatic clash in the dimer may promote the loop opening, while the complementary electrostatic cloud in tetramer(*cccc*) keeps the loop closed. Tetramer(*cocc*) has an intermediate behavior having a positive local electrostatic potential similarly to tetramer(*cccc*) but lower in magnitude.

### Intra-chains contacts variation between tetramer(*cccc*), tetramer(*cocc*), and tetramer(*coco*)

**Fig 7:**
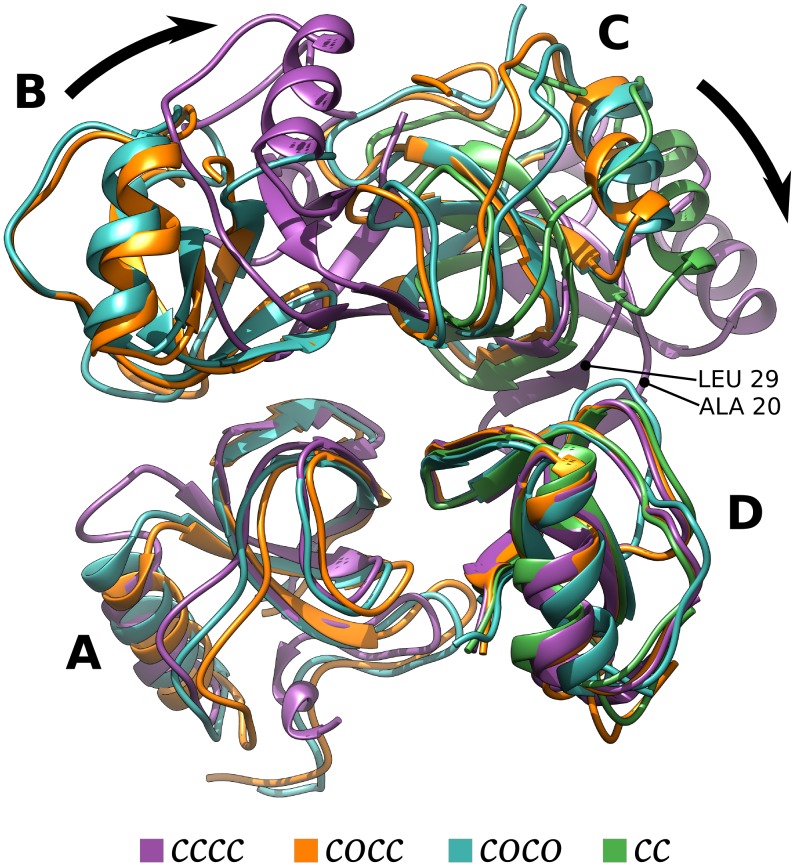
Structural rearrangements. Final structures from standard MD simulations for tetramer(*cccc*), tetramer(*cocc*), tetramer(*coco*), and dimer(*cc*) have been superposed. Chains A and D have been used as reference chains for the superposition. Black arrows indicate the concerned movement of chains B and C in tetramer(*cccc*) compared to the other tetrameric states. The portion of chain C hindering the motion of the *β*-hairpin is labeled by its first (ALA20) and last residue (LEU29).

The importance of long range interactions for the cooperative effect of the loop opening observed in tetramer(*cocc*) is also supported by the analysis of intra-chains interfaces (see S7 Fig and S3 Table). In the tetramer, there are six possible contact interfaces between the four monomeric units. The interfaces between chains A/B and C/D maintained the same area while changing the contact residues, reflecting the *β*-hairpin rearrangement. The interfaces between chains A/D and B/C kept a constant area and the same contact residues. These interfaces were rather rigid, hence, they are not responsible for the cooperative transition. On the other hand, the contact areas between chains A/C and B/D shrank during the transition from tetramer(*cccc*) to tetramer(*coco*) or tetramer(*cocc*). In particular, the interface between B and D, which was already small, disappeared, while interface A/C varied part of its contact residues. The variation of interfaces A/C and B/D depends upon a rigid rotation of the B-C chains with respect to the A-C chains and is not due to local rearrangements, see tetramer(*cccc*) in Fig 7. As a consequence of this rotation, the electrostatic potential at the C/D interface couples with the *β*-hairpin rearrangement, as previously described. Moreover, in tetramer(*cccc*) the opening of the *β*-hairpin (chain D or B) may be hindered by steric effects due to the position of residues 20–29 (chain C or A), see Fig 7.

## Discussion

The goal of this study was to clarify the multimerization mechanism of HFBI in solution. HFBI forms dimers and tetramers in a concentration dependent manner. Above a critical concentration (150 *g*/*L*) HFBI is mainly tetrameric [14, 24]. The crystal structure of HFBI is also a tetramer which contains two types of molecules named in this work *c* and *o* conformations differing only by the position of the last *β*-hairpin motif. The rest of the molecule is exceptionally rigid, due to the presence of four disulfide bridges which stabilize the structure, and is almost identical among the four chains. Using Brownian dynamics simulations, it was found that dimers or tetramers encounter complexes only assemble from *c* conformations [39]. This finding supports the suggestion that the conformational rearrangement of the last *β*-hairpin found in the HFBI crystal structure is induced by tetramer formation [14]. The role of the last *β*-hairpin in the multimerization mechanism was assessed in this work by exploring the transition from conformation *c* to *o* in the monomer, dimer and tetramer using metadynamics. In dimers and tetramers the metadynamic bias was only applied to one monomeric unit, chain D (see Methods for details). Throughout the manuscript, whenever monomer, dimers or tetramers are specified, the conformation of the monomeric units is given in parenthesis and the chain subjected to MetaD is given in bold typeface.

At first, we investigated the preferred conformation of the HFBI monomer in solution. The FES of the monomer obtained from MetaD simulations shows a clear minimum in correspondence of the *c* form (see Figs 2A and 3). In solution, *c* form is thermodynamically favoured. Upon dimerization, multiple minima (mainly three) appear distinctly changing the FES. The minimum in correspondence to the *o* conformation, Figs 2B and 3, is particularly relevant. These results indicate that, in the dimer, the *c* to *o* transition is allowed. In the *c* conformation, the last *β*-hairpin is involved in an anti-parallel *β*-sheet. Several H-bonds must be broken in order to move the *β*-hairpin to the *o* conformation. A possible explanation for the allowed transition within the dimer is the formation of a H-bond network which compensate for the loss of the H-bonds between the last *β*-hairpin and the *β*-sheet. In order to check for this, the H-bond network involving the *β*-hairpin in tetramer(*cccc*) and tetramer(*coco*) has been compared. In *c* conformation, 4.5 hydrogen bonds are present, on average, between the *β*-hairpin and the *β*-sheet (Fig 4A, intra-chain group). In *o* conformation, only transient H-bonds are formed: persistence ≈10% and average number of H-bonds per frame less than 1 in all groups (Fig 4B). The loss of H-bonds is not restored by new H-bonds within the protein. However, looking for H-bonds formed with the solvent, the exposed conformation of *β*-hairpin in *o* form allows several (approximately 6) H-bonds to be established with water molecules, see Fig 4C. If the opening of the *β*-hairpin was due to solvent mediated H-bonds, the monomer in solution could have also been stable in *o* conformation, however this is not observed. The reason for the stabilization of the *o* form in the dimer does not only depend on the H-bonds network. In details, the HFBI monomer has a large non-polar patch exposed to the solvent. The opening of the *β*-hairpin may further increase the non-polar solvent exposed area, thus, destabilizing the molecule. This has been indeed proved by computing the non-polar contribution to the solvation free energy Δ*G*_*non*−*polar*_ for monomer (*c* and *o*), dimer (*cc* and *co*) and tetramers (*cccc* and *coco*). In the monomer the transition from *c* to *o* significantly increases the Δ*G*_*non*−*polar*_ due to the exposure of non-polar residues. In dimers or tetramers, part of the non-polar surface patch is buried by the presence of other monomeric units canceling out the differences between the homo/hetero-dimer and the homo/hetero-tetramer, see Fig 6. This explains why the transition *c*/*o* is allowed in the dimer and not when HFBI is in the monomeric form.

Tetramer formed by four *c* conformations should behave similarly to the dimer, however, the FES of tetramer(*ccc**c***) resembles the monomeric one, see Figs 2C and 3. That is, the equilibrium conformation of the molecule within a homo-tetramer is the *c* form. In tetramer(*ccc**c***) the *β*-hairpin does not undergo a conformational rearrangement due to electrostatic and steric effects. At equilibrium, chains B-C in tetramer(*ccc**c***) rigidly rotate with respect to chains A-D, compared to the tetramer(*coco*) conformation (Fig 7). This rotation leads to a variation of the electrostatic potential at the C/D interface (Fig 5) and to the formation of contacts that reduce the possibility of *β*-hairpin opening (Fig 7 and S3 Table). This coupling between quaternary and tertiary structure rearrangements has been well studied, for example, in hemoglobin [40, 41]. On the other hand, looking at the tetramer(*coc**c***), where one chain is already in conformation *o*, an intermediate behaviour between the monomer and the dimer can be observed in term of FES (Figs 2D and 3). In tetramer(*cocc*), chain C keeps the same internal structure and the same relative orientation with respect to chain D as in the dimer (S6 Fig and Fig 7). However, the electrostatic potential at the C/D interface changes due to the presence of chains A and B as clear from Fig 5 and S2 Video. Changes in the electrostatic potential at the C/D interface are responsible for the lower probability of *β*-hairpin opening in tetramer(*coc**c***). In chain D, the region of the *β*-hairpin has a large negative patch, extending from the molecule surface. On the facing chain (chain C) a region with a negative electrostatic potential is also present, however the magnitude of this negative area changes depending on the presence/absence of chain B and on its conformation. In particular, in the dimer, where chain B is not present, the electrostatic potential has the largest magnitude. In tetramers, when chain B is in *c* conformation, the electrostatic potential at the interface between chain C and the *β*-hairpin is reduced. When chain B is in *o* conformation, the magnitude of negative electrostatic cloud is in between the dimeric and the homo-tetrameric one. The stronger repulsion exists in the dimer where the overlap of the two same-charged regions may promote the opening of the loop. On tetramer(*ccc**c***), the repulsion is notably lower preserving the closed form while in tetramer(*coc**c***) an intermediate behavior occurs, where the opening may happen however with low probability.

**Fig 8:**
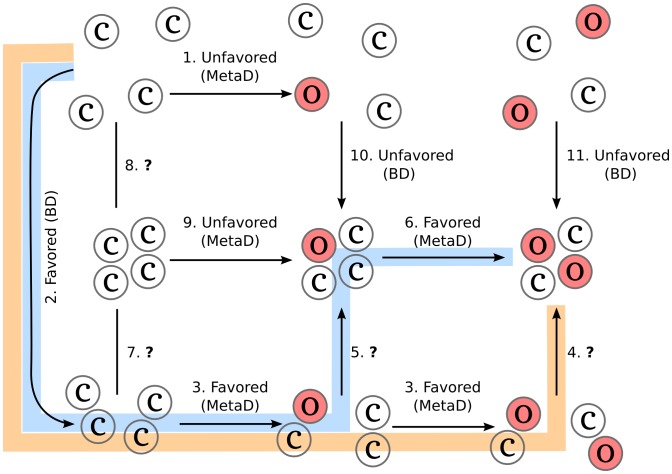
Proposed oligomerization steps. Diagram showing the possible pathways leading to the oligomerization of HFBI in solution. Monomer(*c*) and monomer(*o*) are drawn as empty circles and filled light red circles, respectively. Each connection is labeled as favored or unfavored in a given direction (specified by an arrow) according to what found by metadynamics (MetaD) or Brownian dynamics (BD) simulations. A question mark indicates that not enough information was available to clarify that step. Most probable pathway, given by connections 2-3-4, is highlighted in orange. An alternative pathway formed by connections 2-3-5-6 is highlighted in cyan.

Summarizing these findings, together with the knowledge that dimers and tetramers are present in solution [4, 9], the multimerization mechanism can be dissected, see Fig 8. At first, unfavored routes are excluded. In particular, transition 9 and 10 (see Fig 8) can not occur according to what found by Brownian dynamics simulations [24], and because the monomer(*o*) is not stable in solution as found by MetaD simulations. We do not have enough information to determine the preferred direction of transition 7 and 8. From Brownian dynamics simulations, tetramers in *c* forms have been observed, however, it is highly likely that they are transient encounter complexes. This idea is supported by the unfavored transition from *c* to *o* within a tetramer(*cccc*) found in this work. Two possible multimerization mechanism can now be proposed.

The first one, the most probable route, implies the association of two monomers in *c* conformation into a dimer *cc*, transition 2 in Fig 8. This association is supported by Brownian dynamics simulations results [24] where dimeric *cc* encounter complexes were found to be favoured over *co* and almost no dimers in *oo* conformation were found. Within the dimer, the conformational change of one molecule to *o* (transitions 3) is largely favoured according to our findings. Then, it is possible that two *co* dimers can now assembly into stable tetramer(*coco*) (transition 4). No direct evidence is available for this last step, however, this route is consistent with the general finding that passing through dimers is evolutionary preferred [9, 23]. Another possible route, is the association of one *co* dimer with one *cc* dimer into a tetramer(*cocc*) (transition 5). Then, the motion of the last chain to *o* conformation can occur according to the results of MetaD simulations (transition 6). This second mechanism is less probable compared to the first one as the *cocc* → *coco* transition is not as favoured as in the dimer. The small free energy differences in dimer(*co*) and tetramer(*coco*) imply that the *β*-hairpin can relatively easily go back and forth between the *c* and the *o* conformation. This can be also seen looking at the densities of the conformational states of tetramer(*coco*) in standard MD simulations. Already in 300 ns, the *β*-hairpin performs large movements passing by $\delta_{{min}_{3}}\;=\;1.25\text{ nm}$, $\delta_{{min}_{2}}\;=\;0.81\text{ nm}$, and $\delta_{{min}_{1}}\;=\;0.47\text{ nm}$ on the distance coordinate. A complete transition from *o* to *c*, which implies the rotation of *τ*, is not however observed in standard MD.

These findings allow to draw a biological role for the proposed association mechanism. As previously suggested [20], hydrophobin multimerization is an efficient way to protect the large hydrophobic patch, i.e. avoid unwanted strong unspecific interactions. Nevertheless, in order to exploit their biological function (e.g. lowering the water surface tension while the hyphae are growing [16]), multimers must not be overly stable: they have to dissociate at the air/water interface [9, 20]. The motion of the last *β*-hairpin is essential to fine tune the stability of the HFBI multimers. It is highly likely that the arrangement of HFBI at the interfaces is also affected, as the hydrophobic interaction surface and lateral interactions are modified by the movement of the last *β*-hairpin. This result is remarkably important in order to clarify the mechanism of arranging at the interface and enhancing hydrophobin-based technological applications [42]. More generally, the strategy where a rigid patch flanked by a flexible region allows to adjust protein-protein interaction energy, was already found in other protein complexes [43]. However, the interface was composed of charged residues [43, 44]. To the best of our knowledge this is the first example where this unique fine-tuning association mechanism occurs within a hydrophobic interface.

## Supporting Information

S1 Table Summary of the simulation setup and the conformational sampling of the single monomeric units. ^*a*^: standard molecular dynamics simulations (MD) or well-tempered metadynamics (MetaD). ^*b*^: Na^+^ and Cl^−^ were added to neutralize the system and to reach a ionic concentration of 150 mM. ^*c*^: for MetaD, the simulations were run till convergence as explained in Materials and Methods section. ^*d*^: for standard MD simulations this refers to the conformations of the final snapshots, while for MetaD it refers to the conformations sampled during the whole simulation. ^*e*^: each monomeric unit is separated by |.
(PDF)

S2 Table Hydrogen bonds formed by the aminoacids in the *β*-hairpin and the rest of the molecule. The intra-hairpin includes hydrogen bonds formed within the residues 60–66 forming the *β*-hairpin. The intra-chain group corresponds to the hydrogen-bonds between the *β*-hairpin and the rest of the chain. The inter-chain group contains hydrogen-bonds established between the *β*-hairpin and the chain facing the *β*-hairpin. H-bonds analysis was performed on one monomeric unit (chain D) within tetramer(*cccc*) as well as tetramer(*coco*) using all frames of the 300 ns standard MD simulations. Donor/acceptor atoms names according to the AMBER force field.
(PDF)

S3 Table Residues at the interfaces. List of interacting residues defined as pairs of aminoacids whose average (over time) smallest distance (among heavy atoms only) was up to 0.45 nm, see Methods for details. The interface area for each tetrameric conformation (*cccc*, *cocc*, and *coco*) is also indicated, in brackets the 95% confidence interval is given.
(PDF)

S1 Video Dimer metadynamics video. Video showing the metadynamics of the dimer. Metadynamic bias have been applied to the *β*-hairpin in chain D (bottom monomeric unit). During the simulation, the *β*-hairpin opens and closes repeatedly. Trajectory has been smoothed for better visualization.
(MP4)

S2 Video Electrostatic potential morphing. In order to visually appreciate the differences between the electrostatic potential of *ccc* and *coc*, a morphing transition between the 3D electrostatic potential maps has been computed using the Chimera software [38]. Red regions show negative potential while blue regions indicate positive potential. For a clearer visualization the movie shows the transition from *ccc* to *coc* and then back to *ccc*. It is clearly visible the negative cloud growing from *ccc* to *coc* in the region of the last *β*-hairpin at the interface with chain D (pink ribbon).
(MP4)

S1 Fig Secondary structure and disulphide bonds. Secondary structure was calculated with DSSP [45] on the Protein Data Bank website (www.rcsb.org/pdb). Here, we report the results for each monomeric unit (chains A-B-C-D) of the crystal structure of HBFI (PDB id: 2FZ6).
(TIFF)

S2 Fig Starting conformations. Ribbon representation of the structure of monomers/dimer/tetramers used as initial conformations in standard MD or MetaD.
(TIFF)

S3 FigMetaD convergence analysis.Time dependent free energy profiles on *δ* computed on successive blocks ([0, i*10*ns*] intervals) are overlapped. Convergence is achieved when the last blocks overlap. The colorbar indicates the blocks progression. This analysis has been repeated for the monomer(***c***) (A1), dimer(*c**c***) (B1), tetramer(*ccc**c***) (C1) and tetramer(*coc**c***) (D1). Moreover, the free energy value at $\delta_{{min}_{3}}\;=\;1.25\text{ nm}$ have been obtained from the *i*^*th*^ profile. Then, the root mean square of two consecutive profiles is computed. $RMS\left(\Delta G_{min3}\left(\delta \right)\right)=\sqrt{\left[\Delta G_{\left(i\right)}^{min3}\left(\delta \right)-\Delta G_{\left(i-1\right)}^{Min3}\left(\delta \right)\right]}$. Blue line is a local regression fit (loess [46]), gray shadows confidence intervals from loess fit. Monomer(***c***) (A2), dimer(*c**c***) (B2), tetramer(*ccc**c***) (C2) and tetramer(*coc**c***) (D2).(TIFF)

S4 Fig Joint probability density functions. Joint probability density functions obtained from metadynamics simulations on the collective variables *δ* and *τ* for (A) Monomer(***c***), (B) Dimer(*c**c***), (C) Tetramer(*ccc**c***), and (D) Tetramer(*coc**c***). Superposed contour lines define regions which enclose 90% of the conformations sampled by the *c* (continuous) and *o* (dashed) form during 300 ns standard MD simulations.
(TIFF)

S5 Fig Scatter plot for collective variables. Values of *δ* and *τ* are shown for chains A, B, and C in tetramer(*ccc**c***) and tetramer(*coc**c***) from MetaD simulations. Superposed contour lines define regions which enclose 90% of the conformations sampled by the *c* (continuous) and *o* (dashed) form during 300 ns standard MD simulations. As visible from the scatter plot, chains A, B, and C, which have not been subjected to the MetaD bias, remain in their initial conformation.
(TIFF)

S6 Fig Chain C comparison. The root mean squared fluctuation (RMSF, i.e. the standard deviation) of atomic positions was computed for chain C from tetramer(*cccc*), tetramer(*coco*), and dimer(*cc*) from standard MD simulations. From the atomic RMSF the residue average is computed. Trajectories have been split into 5 blocks and the RMSF was computed on each block. On top, the RMSF profiles are shown as mean ± standard error of the mean, n = 5. The average difference between the three RMSF profiles expressed as root mean square is 0.07 nm. Bottom, the structures (last trajectory frame) of chain C in tetramer(*cccc*) (purple), tetramer(*coco*) (green) and dimer(*cc*) (cyan) have been superposed. The root mean square deviation (RMSD) between the atomic position of tetramer(*cccc*) or dimer(*cc*) and tetramer(*coco*) is less than 0.15 nm (hydrogens excluded).
(TIFF)

S7 Fig Distance map. Residues distance maps computed for the chain pairs A/B, A/C, A/D, B/C, B/D, and C/D within tetramer(*cccc*), tetramer(*cocc*), and tetramer(*coco*). Distance maps have been obtained by measuring the smallest distances between residue pairs (heavy atoms only) for all trajectory frames and averaging over time. Analyses have been performed on standard MD simulations. See Method Section for further details.
(TIFF)

## References

[1] Aimanianda V, Bayry J, Bozza S, Kniemeyer O, Perruccio K, Elluru SR, et al Surface hydrophobin prevents immune recognition of airborne fungal spores. Nature. 2009;460(7259):1117–1121. 10.1038/nature08264 19713928

[2] Linder MB. Hydrophobins: Proteins that self assemble at interfaces. Current Opinion in Colloid & Interface Science. 2009;14(5):356–363. 10.1016/j.cocis.2009.04.001

[3] Talbot NJ. Fungal biology. Coming up for air and sporulation. Nature. 1999;398(6725):295–296. 10.1038/18575 10192329

[4] Szilvay GR, Paananen A, Laurikainen K, Vuorimaa E, Lemmetyinen H, Peltonen J, et al Self-assembled hydrophobin protein films at the air-water interface: structural analysis and molecular engineering. Biochemistry. 2007;46(9):2345–2354. 10.1021/bi602358h 17297923

[5] Hektor HJ, Scholtmeijer K. Hydrophobins: proteins with potential. Current opinion in biotechnology. 2005;16(4):434–9. 10.1016/j.copbio.2005.05.004 15950452

[6] Sunde M, Kwan AHY, Templeton MD, Beever RE, Mackay JP. Structural analysis of hydrophobins. Micron. 2008;39(7):773–784. 10.1016/j.micron.2007.08.003 17875392

[7] Wösten Ha. Hydrophobins: multipurpose proteins. Annual review of microbiology. 2001;55:625–646. 10.1146/annurev.micro.55.1.625 11544369

[8] Lienemann M, Gruner MS, Paananen A, Siika-aho M, Linder MB. Charge-Based Engineering of Hydrophobin HFBI: Effect on Interfacial Assembly and Interactions. Biomacromolecules. 2015;16:1283–1292. 10.1021/acs.biomac.5b00073 25724119

[9] Szilvay GR, Nakari-Setälä T, Linder MB. Behavior of Trichoderma reesei hydrophobins in solution: interactions, dynamics, and multimer formation. Biochemistry. 2006;45(28):8590–8598. 10.1021/bi060620y 16834333

[10] Valo HK, Laaksonen PH, Peltonen LJ, Linder MB, Hirvonen JT, Laaksonen TJ. Multifunctional Hydrophobin: Toward Nanoparticles. ACS Nano. 2010;4(3):17501758 10.1021/nn9017558 20210303

[11] Cox AR, Aldred DL, Russell AB. Exceptional stability of food foams using class II hydrophobin HFBII. Food Hydrocolloids. 2009;23(2):366–376. 10.1016/j.foodhyd.2008.03.001

[12] Lumsdon SO, Green J, Stieglitz B. Adsorption of hydrophobin proteins at hydrophobic and hydrophilic interfaces. Colloids and surfaces B, Biointerfaces. 2005;44(4):172–178. 10.1016/j.colsurfb.2005.06.012 16085399

[13] Peng C, Liu J, Zhao D, Zhou J. Adsorption of Hydrophobin on Different Self-Assembled Monolayers: The Role of the Hydrophobic Dipole and the Electric Dipole. Langmuir. 2014;30:11401–11411. 10.1021/la502595t 25185838

[14] Szilvay GR, Kisko K, Serimaa R, Linder MB. The relation between solution association and surface activity of the hydrophobin HFBI from Trichoderma reesei. FEBS letters. 2007;581(14):2721–2726. 10.1016/j.febslet.2007.05.024 17531982

[15] Kisko K, Szilvay GR, Vainio U, Linder MB, Serimaa R. Interactions of hydrophobin proteins in solution studied by small-angle X-ray scattering. Biophysical journal. 2008;94(1):198–206. 10.1529/biophysj.107.112359 17827247 PMC2134873

[16] Wessels JGH. Developmental Regulation of Fungal Cell Wall Formation. Annual Review of Phytopathology. 1994;32(1):413–437. 10.1146/annurev.py.32.090194.002213

[17] Kwan AHY, Winefield RD, Sunde M, Matthews JM, Haverkamp RG, Templeton MD, et al Structural basis for rodlet assembly in fungal hydrophobins. PNAS. 2006;103(10):3621–3626. 10.1073/pnas.0505704103 16537446 PMC1533775

[18] Paananen A, Vuorimaa E, Torkkeli M, Penttila M, Kauranen M, Ikkala O, et al Structural hierarchy in molecular films of two class II hydrophobins. Biochemistry. 2003;42:5253–5258. 10.1021/bi034031t 12731866

[19] Torkkeli M, Serimaa R, Ikkala O, Linder M. Aggregation and Self-Assembly of Hydrophobins from Trichoderma reesei: Low-Resolution Structural Models. Biophysical journal. 2002;83(4):2240–2247. 10.1016/S0006-3495(02)73984-2 12324441 PMC1302312

[20] Hakanpää J, Szilvay GR, Kaljunen H, Maksimainen M, Linder M, Rouvinen J. Two crystal structures of Trichoderma reesei hydrophobin HFBI–the structure of a protein amphiphile with and without detergent interaction. Protein science. 2006;15(9):2129–2140. 10.1110/ps.062326706 16882996 PMC2242604

[21] Kisko K, Szilvay GR, Vuorimaa E, Lemmetyinen H, Linder MB, Torkkeli M, et al Self-assembled films of hydrophobin proteins HFBI and HFBII studied in situ at the air/water interface. Langmuir. 2009;25(3):1612–1619. 10.1021/la803252g 19093751

[22] Krivosheeva O, Deìdinaiteì A, Linder MB, Tilton RD, Claesson PM. Kinetic and equilibrium aspects of adsorption and desorption of class II hydrophobins HFBI and HFBII at silicon oxynitride/water and air/water interfaces. Langmuir. 2013;29(8):2683–2691. 10.1021/la3048888 23356719

[23] Powers ET, Powers DL. A perspective on mechanisms of protein tetramer formation. Biophysical journal. 2003;85(6):3587–3599. 10.1016/S0006-3495(03)74777-8 14645052 PMC1303664

[24] Mereghetti P, Wade RC. Diffusion of hydrophobin proteins in solution and interactions with a graphite surface. BMC Biophysics. 2011;4(9):2–11. 10.1186/2046-1682-4-2 21595866 PMC3114038

[25] Bussi G, Donadio D, Parrinello M. Canonical sampling through velocity rescaling. The Journal of Chemical Physics. 2007;126:014101 10.1063/1.2408420 17212484

[26] Parrinello M, Rahman A. Polymorphic transitions in single crystals: A new molecular dynamics method. Journal of Applied Physics. 1981;52:7182–7190. 10.1063/1.328693.

[27] Hess B, Bekker H, Berendsen HJC, Fraaije JGEM. LINCS: A linear constraint solver for molecular simulations. Journal of Computational Chemistry. 1997;18:1463–1472. 10.1002/(SICI)1096-987X(199709)18:12%3C1463::AID-JCC4%3E3.0.CO;2-H

[28] Darden T, York D, Pedersen L. Particle mesh Ewald: An Nlog(N) method for Ewald sums in large systems. The Journal of Chemical Physics. 1993;98(12):10089–10092. 10.1063/1.464397

[29] Abraham MJ, Murtola T, Schulz R, Páll S, Smith JC, Hess B, et al GROMACS: High performance molecular simulations through multi-level parallelism from laptops to supercomputers. SoftwareX. 2015;1–2:19–25. 10.1016/j.softx.2015.06.001

[30] Lindorff-Larsen K, Piana S, Palmo K, Maragakis P, Klepeis JL, Dror RO, et al Improved side-chain torsion potentials for the Amber ff99SB protein force field. Proteins: Structure, Function, and Bioinformatics. 2010;78(8):1950–1958. 10.1002/prot.22711 20408171 PMC2970904

[31] Laio A, Gervasio FL. Metadynamics: a method to simulate rare events and reconstruct the free energy in biophysics, chemistry and material science. Reports on Progress in Physics. 2008;71(12):126601 10.1088/0034-4885/71/12/126601

[32] Tribello GA, Bonomi M, Branduardi D, Camilloni C, Bussi G. {PLUMED} 2: New feathers for an old bird. Computer Physics Communications. 2014;185(2):604–613. 10.1016/j.cpc.2013.09.018

[33] Kumari R, Kumar R, Source O, Discovery D, Lynn A. g_mmpbsa—A GROMACS Tool for High-Throughput MM-PBSA Calculations. Journal of chemical information and modeling. 2014;54:1951–1962. 10.1021/ci500020m 24850022

[34] Baker NA, Sept D, Joseph S, Holst MJ, McCammon JA. Electrostatics of nanosystems: Application to microtubules and the ribosome. Proceedings of the National Academy of Sciences. 2001;98(18):10037–10041. 10.1073/pnas.181342398 11517324 PMC56910

[35] Rashid Q, Kapil C, Singh P, Kumari V, Aman Jairajpuri M. Understanding the specificity of serpin-protease complexes through interface analysis. Journal of biomolecular structure & dynamics. 2014;(July):1–37. 10.1080/07391102.2014.947525 25052369

[36] R Core Team. R: A Language and Environment for Statistical Computing; 2015 Available from: https://www.R-project.org/.

[37] Humphrey W, Dalke A, Schulten K. VMD—Visual Molecular Dynamics. Journal of Molecular Graphics. 1996;14:33–38. 8744570 10.1016/0263-7855(96)00018-5

[38] Pettersen EF, Goddard TD, Huang CC, Couch GS, Greenblatt DM, Meng EC, et al UCSF Chimera—A visualization system for exploratory research and analysis. Journal of Computational Chemistry. 2004;25(13):1605–1612. 10.1002/jcc.20084 15264254

[39] Mereghetti P, Gabdoulline RR, Wade RC. Brownian dynamics simulation of protein solutions: structural and dynamical properties. Biophys J. 2010;99(11):3782–3791. 10.1016/j.bpj.2010.10.035 21112303 PMC2998633

[40] Vesper MD, de Groot BL. Collective Dynamics Underlying Allosteric Transitions in Hemoglobin. PLoS Computational Biology. 2013;9(9). 10.1371/journal.pcbi.1003232 24068910 PMC3777908

[41] Yusuff OK, Babalola JO, Bussi G, Raugei S. Role of the Subunit Interactions in the Conformational Transitions in Adult Human Hemoglobin: An Explicit Solvent Molecular Dynamics Study. The Journal of Physical Chemistry B. 2012;116(36):11004–11009. 10.1021/jp3022908 22838506

[42] Soikkeli M, Kurppa K, Kainlauri M, Arpiainen S, Paananen A, Gunnarsson D, et al Graphene biosensor programming with genetically engineered fusion protein monolayers. ACS Applied Materials & Interfaces. 2016; 10.1021/acsami.6b00123 26960769

[43] Wei G, Xi W, Nussinov R, Ma B. Protein Ensembles: How Does Nature Harness Thermodynamic Fluctuations for Life? The Diverse Functional Roles of Conformational Ensembles in the Cell. Chemical Reviews. 2016;XXX:XXX. 10.1021/acs.chemrev.5b00562 26807783 PMC6407618

[44] Ma B, Elkayam T, Wolfson H, Nussinov R. Protein–protein interactions: Structurally conserved residues distinguish between binding sites and exposed protein surfaces. Proceedings of the National Academy of Sciences. 2003;100(10):5772–5777. 10.1073/pnas.1030237100 12730379 PMC156276

[45] Kabsch W, Sander C. Dictionary of protein secondary structure: Pattern recognition of hydrogen-bonded and geometrical features. Biopolymers. 1983;22(12):2577–2637. 10.1002/bip.360221211 6667333

[46] Cleveland WS. Robust Locally Weighted Regression and Smoothing Scatterplots. Journal of the American Statistical Association. 1979;74(368):829–836. 10.1080/01621459.1979.10481038

