# Supplementary material for: Induced Fit in Protein Multimerization: The HFBI Case
Source: PLoS Comput Biol. 2016 Nov 10;12(11):e1005202. doi: 10.1371/journal.pcbi.1005202 (PMC5104427; doi:10.1371/journal.pcbi.1005202)
Supplement: S1 Table — a: standard molecular dynamics simulations (MD) or well-tempered metadynamics (MetaD). b: Na+ and Cl− were added to neutralize the system and to reach a ionic concentration of 150 mM. c: for MetaD, the simulations were run till convergence as explained in Materials and Methods section. d: for standard MD simulations this refers to the conformations of the final snapshots, while for MetaD it refers to the conformations sampled during the whole simulation. e: each monomeric unit is separated by |. (PDF) [file pcbi.1005202.s001.pdf]

S1 Table.

Summary of the simulation setup and the conformational sampling of the single monomeric units. [a]: standard molecular dynamics simulation (MD) or well-tempered metadynamics (MetaD). [b]: Na^+ and Cl^- were added to neutralize the system and to reach a ionic concentration of 150 m. [c]: for MetaD, the simulation were run till convergence as explained in Materials and Methods section. [d]: for standard MD simulation this refers to the conformations of the final snapshots, while for MetaD it refers to the conformations sampled during the whole simulation. [e]: each monomeric unit is separated by |.

System	Method[a]	Water molecules	Ions[b] Zn^{2+}	Na^+	Cl^-	Total # atoms	Time[c] (ns)	Visited[d,e] conformations
monomer(c)	MD	3390	-	10	10	11192	150	c/c
monomer(o)	MD	3510	-	11	11	11515	150	o/o
dimer(cc)	MD	10048	1	29	31	10048	100	$c/c\|c/c$
tetramer($cccc$)	MD	12167	2	35	39	40585	300	$c/c\|c/c\|c/c\|c/c$
tetramer($cocc$)	MD	12167	2	35	39	40585	100	$c/c\|o/o\|c/c\|c/c$
tetramer($coco$)	MD	12492	2	36	40	41470	300	$c/c\|o/o\|c/c\|o/o$
monomer($\boldsymbol{c}$)	MetaD	3390	-	10	10	11192	200	c/c
dimer($c\boldsymbol{c}$)	MetaD	10048	1	29	31	10048	200	$c/c\|c/o$
tetramer($ccc\boldsymbol{c}$)	MetaD	12167	2	35	39	40585	200	$c/c\|c/c\|c/c\|c/c$
tetramer($coc\boldsymbol{c}$)	MetaD	12179	2	35	39	40582	200	$c/c\|o/o\|c/c\|c/o$
