# Supplementary material for: Induced Fit in Protein Multimerization: The HFBI Case
Source: PLoS Comput Biol. 2016 Nov 10;12(11):e1005202. doi: 10.1371/journal.pcbi.1005202 (PMC5104427; doi:10.1371/journal.pcbi.1005202)
Supplement: S2 Table — The intra-hairpin includes hydrogen bonds formed within the residues 60–66 forming the β-hairpin. The intra-chain group corresponds to the hydrogen-bonds between the β-hairpin and the rest of the chain. The inter-chain group contains hydrogen-bonds established between the β-hairpin and the chain facing the β-hairpin. H-bonds analysis was performed on one monomeric unit (chain D) within tetramer(cccc) as well as tetramer(coco) using all frames of the 300 ns standard MD simulations. Donor/acceptor atoms names according to the AMBER force field. (PDF) [file pcbi.1005202.s002.pdf]

S2 Table.

Hydrogen bonds formed by the aminoacids in the β-hairpin and the rest of the molecule. The intra-hairpin includes hydrogen bonds formed within the residues 60-66 forming the β-hairpin. The intra-chain group correspond to the hydrogen-bonds between the β-hairpin and the rest of the chain. The inter-chain group contains hydrogen-bonds established between the β-hairpin and the chain facing the β-hairpin. H-bonds analysis was performed on one monomeric unit (chain D) within tetramer(*cccc*) as well as tetramer(*coco*) using all frames of the 300 ns standard MD simulations. Donor/acceptor atoms names according to the AMBER force field.

Tetramer(*cccc*)

Type	Donor			Acceptor			Persistence
	Res. name	Res n.	Atom	Res. name	Res n.	Atom	
Intra-hairpin	GLN	65	N	GLN	65	OE1	0.03
Intra-chain	CYS	31	N	GLY	64	O	0.65
Intra-chain	ASP	30	N	GLN	65	O	0.99
Intra-chain	GLN	17	NE2	VAL	62	O	0.90
Intra-chain	GLN	17	NE2	ALA	60	O	0.70
Intra-chain	ASN	2	ND2	PRO	61	O	0.27
Intra-chain	GLN	65	NE2	ASP	30	OD2	0.05
Intra-chain	GLN	65	NE2	ASP	30	OD1	0.04
Intra-chain	GLY	64	N	CYS	31	O	0.89
Inter-chain	GLN	65	NE2	GLN	65	O	0.49
Inter-chain	GLN	65	NE2	GLN	65	OE1	0.11

Tetramer(*coco*)

Type	Donor			Acceptor			Persistence
	Res. name	Res n.	Atom	Res. name	Res n.	Atom	
Intra-hairpin	ALA	66	N	GLN	65	OE1	0.03
Intra-hairpin	GLN	65	NE2	GLY	64	O	0.03
Intra-hairpin	GLN	65	NE2	ALA	63	O	0.06
Intra-hairpin	GLN	65	N	ALA	63	O	0.08
Intra-hairpin	GLN	65	N	VAL	62	O	0.32
Intra-chain	CYS	31	N	GLN	65	O	0.06
Intra-chain	GLY	64	N	CYS	31	O	0.06
Inter-chain	THR	21	OG1	GLN	65	OE1	0.03
Inter-chain	THR	21	OG1	GLY	64	O	0.15
Inter-chain	ALA	66	N	THR	21	OG1	0.10
Inter-chain	GLN	65	NE2	GLY	52	O	0.03
