# Supplementary material for: Induced Fit in Protein Multimerization: The HFBI Case
Source: PLoS Comput Biol. 2016 Nov 10;12(11):e1005202. doi: 10.1371/journal.pcbi.1005202 (PMC5104427; doi:10.1371/journal.pcbi.1005202)
Supplement: S3 Table — List of interacting residues defined as pairs of aminoacids whose average (over time) smallest distance (among heavy atoms only) was up to 0.45 nm, see Methods for details. The interface area for each tetrameric conformation (cccc, cocc, and coco) is also indicated, in brackets the 95% confidence interval is given. (PDF) [file pcbi.1005202.s003.pdf]

S3 Table.

Residues at the interfaces. List of interacting residues defined as pairs of aminoacids whose average (over time) smallest distance (among heavy atoms only) was up to 0.45 nm, see Methods for details. The interface area for each tetrameric conformation (*cccc*, *cocc*, and *coco*) is also indicated, in brackets the 95% confidence interval are given.

Interface A-B

cccc	*cocc*	*coco*
6.8[6.1-7.5] nm^2	6.6[5.8-7.3] nm^2	6.8[5.9-7.7] nm^2
I27(A)-I27(B)	-	-
L29(A)-I27(B)	L29(A)-I27(B)	L29(A)-I27(B)
Q65(A)-L29(B)	Q65(A)-L29(B)	Q65(A)-L29(B)
A66(A)-L29(B)	-	-
Q65(A)-D30(B)	-	-
D30(A)-Q65(B)	-	-
Q65(A)-Q65(B)	-	-
L29(A)-A66(B)	-	-
Q65(A)-A66(B)	-	-
L29(A)-L67(B)	-	-
V23(A)-L68(B)	-	-
-	Q65(A)-T21(B)	Q65(A)-T21(B)
-	L29(A)-V23(B)	L29(A)-V23(B)
-	Q65(A)-V23(B)	Q65(A)-V23(B)
-	L29(A)-L29(B)	L29(A)-L29(B)
-	D30(A)-L29(B)	D30(A)-L29(B)
-	D30(A)-D30(B)	D30(A)-D30(B)
-	T21(A)-G64(B)	T21(A)-G64(B)
-	T21(A)-Q65(B)	T21(A)-Q65(B)
-	T21(A)-A66(B)	T21(A)-A66(B)
-	-	V23(A)-I27(B)

Interface C-D

cccc	*cocc*	*coco*
6.4[5.6-7.3] nm^2	6.9[6.3-7.6] nm^2	6.7[5.8-7.6] nm^2
I27(C)-I27(D)	-	-
L29(C)-I27(D)	L29(C)-I27(D)	L29(C)-I27(D)
Q65(C)-L29(D)	Q65(C)-L29(D)	Q65(C)-L29(D)
D30(C)-Q65(D)	D30(C)-Q65(D)	-
Q65(C)-Q65(D)	-	-
L29(C)-A66(D)	-	-
Q65(C)-A66(D)	-	-
V23(C)-L68(D)	-	-
-	Q65(C)-T21(D)	Q65(C)-T21(D)
-	L29(C)-V23(D)	L29(C)-V23(D)
-	Q65(C)-V23(D)	Q65(C)-V23(D)
-	A66(C)-V23(D)	-
-	D30(C)-L29(D)	D30(C)-L29(D)
-	D30(C)-D30(D)	D30(C)-D30(D)
-	A20(C)-Q65(D)	-
-	G52(C)-Q65(D)	-
-	T21(C)-A66(D)	T21(C)-A66(D)
-	-	L29(C)-L29(D)
-	-	T21(C)-G64(D)
-	-	T21(C)-Q65(D)

Interface A-C

cccc	*cocc*	*coco*
4.7[3.8-5.3] nm^2	3.3[2.8-3.8] nm^2	3.4[2.8-3.9] nm^2
L26(A)-L24(C)	L26(A)-L24(C)	L26(A)-L24(C)
L68(A)-L24(C)	-	-
L24(A)-L26(C)	L24(A)-L26(C)	L24(A)-L26(C)
L26(A)-L26(C)	-	-
L24(A)-L68(C)	-	-
I27(A)-L68(C)	-	-
-	L24(A)-L24(C)	-
-	I27(A)-L24(C)	I27(A)-L24(C)
-	L24(A)-I27(C)	L24(A)-I27(C)
-	I27(A)-I27(C)	I27(A)-I27(C)

Interface A-D

cccc	*cocc*	*coco*
5.6(1.0) nm^2	5.8(0.9) nm^2	5.4(0.8) nm^2
I27(A)-L24(D)	I27(A)-L24(D)	I27(A)-L24(D)
V59(A)-L24(D)	V59(A)-L24(D)	V59(A)-L24(D)
A66(A)-L24(D)	A66(A)-L24(D)	A66(A)-L24(D)
L67(A)-L24(D)	L67(A)-L24(D)	L67(A)-L24(D)
L68(A)-L24(D)	L68(A)-L24(D)	L68(A)-L24(D)
V59(A)-G25(D)	V59(A)-G25(D)	V59(A)-G25(D)
L67(A)-G25(D)	L67(A)-G25(D)	L67(A)-G25(D)
L12(A)-L26(D)	L12(A)-L26(D)	L12(A)-L26(D)
V59(A)-L26(D)	V59(A)-L26(D)	V59(A)-L26(D)
L68(A)-L26(D)	L68(A)-L26(D)	L68(A)-L26(D)
-	A66(A)-G25(D)	A66(A)-G25(D)
-	-	A60(A)-G25(D)

Interface B-C

cccc	*cocc*	*coco*
6.4[5.2-7.5] nm^2	5.3[4.4-6.2] nm^2	5.3[4.5-6.2] nm^2
L26(B)-L12(C)	L26(B)-L12(C)	L26(B)-L12(C)
L24(B)-I27(C)	L24(B)-I27(C)	L24(B)-I27(C)
L24(B)-V59(C)	L24(B)-V59(C)	L24(B)-V59(C)
G25(B)-V59(C)	G25(B)-V59(C)	G25(B)-V59(C)
L26(B)-V59(C)	L26(B)-V59(C)	L26(B)-V59(C)
G25(B)-A60(C)	-	-
L24(B)-A66(C)	L24(B)-A66(C)	L24(B)-A66(C)
L24(B)-L67(C)	L24(B)-L67(C)	L24(B)-L67(C)
G25(B)-L67(C)	G25(B)-L67(C)	G25(B)-L67(C)
L24(B)-L68(C)	L24(B)-L68(C)	L24(B)-L68(C)
L26(B)-L68(C)	L26(B)-L68(C)	L26(B)-L68(C)
-	G25(B)-A66(C)	G25(B)-A66(C)
-	-	F13(B)-L12(C)

Interface B-D

cccc	*cocc*	*coco*
2.3[1.4-3.2] nm^2	0.4[0.2-0.6) nm^2	0.4[0.2-0.6] nm^2
L24(B)-L24(D)	-	-
