# Supplementary figures and images for: Induced Fit in Protein Multimerization: The HFBI Case
Source: PLoS Comput Biol. 2016 Nov 10;12(11):e1005202. doi: 10.1371/journal.pcbi.1005202 (PMC5104427; doi:10.1371/journal.pcbi.1005202)

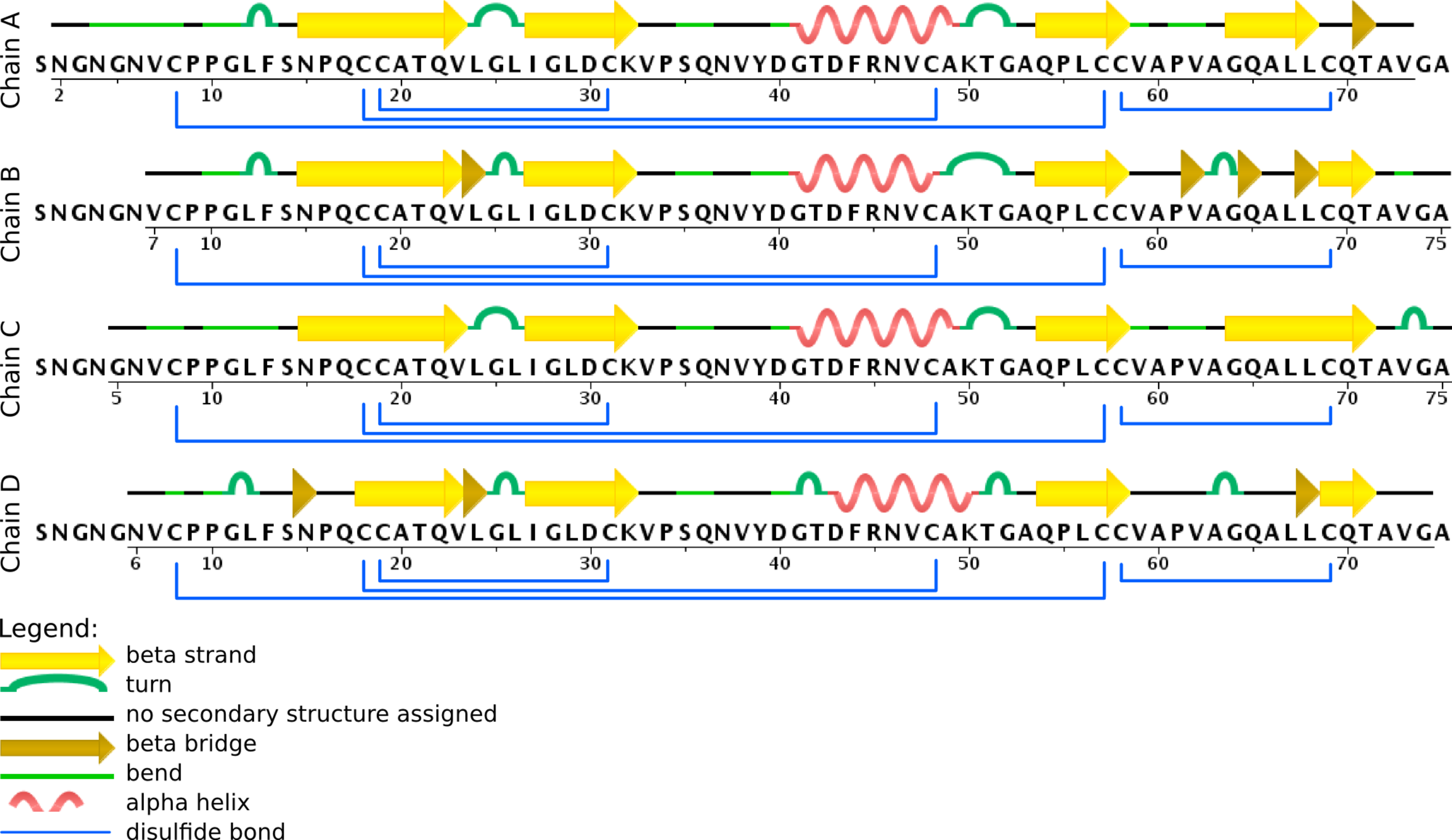

Supplement: S1 Fig — Secondary structure was calculated with DSSP [45] on the Protein Data Bank website (www.rcsb.org/pdb). Here, we report the results for each monomeric unit (chains A-B-C-D) of the crystal structure of HBFI (PDB id: 2FZ6). (TIFF) [file pcbi.1005202.s006.tiff]

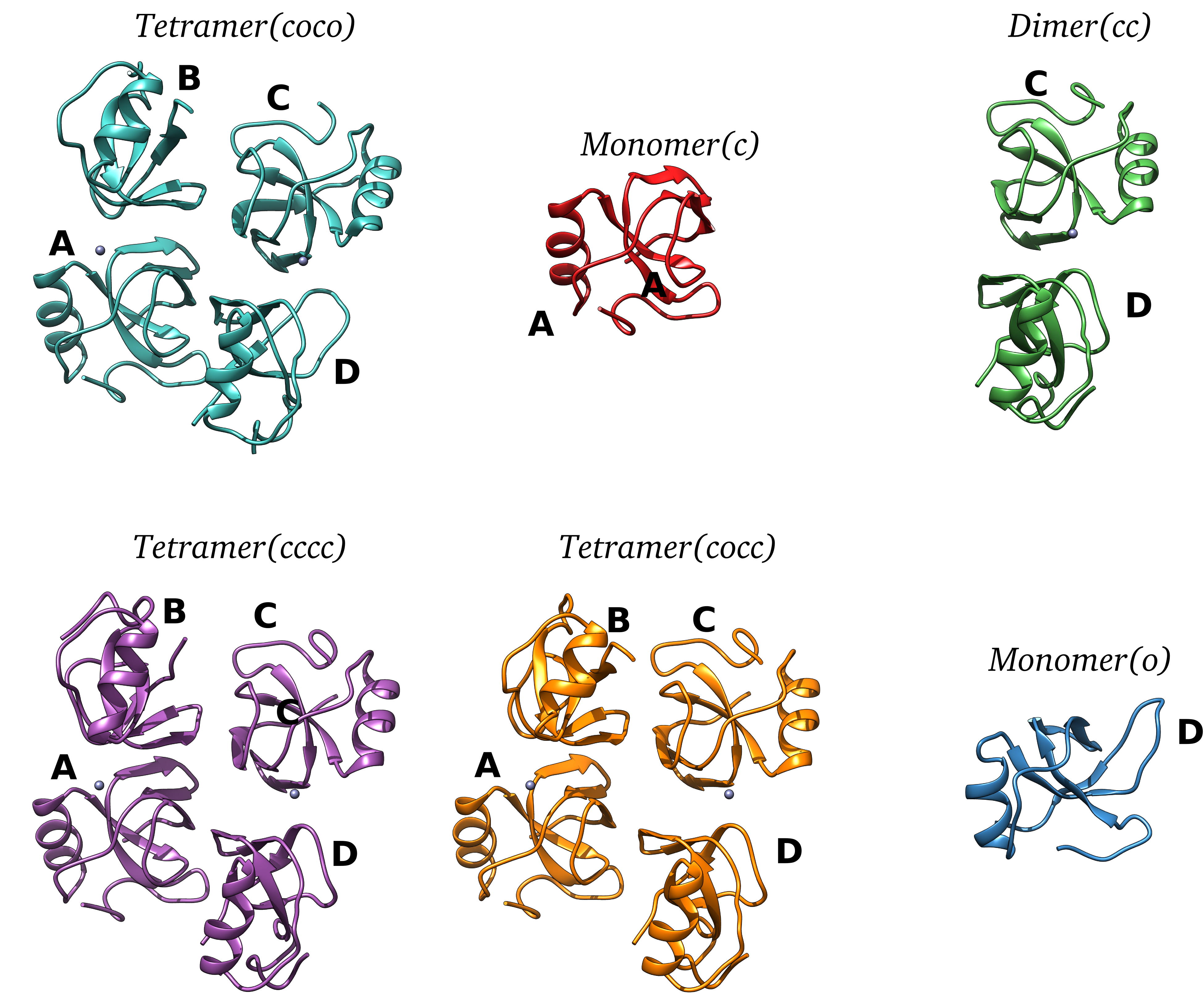

Supplement: S2 Fig — Ribbon representation of the structure of monomers/dimer/tetramers used as initial conformations in standard MD or MetaD. (TIFF) [file pcbi.1005202.s007.tiff]

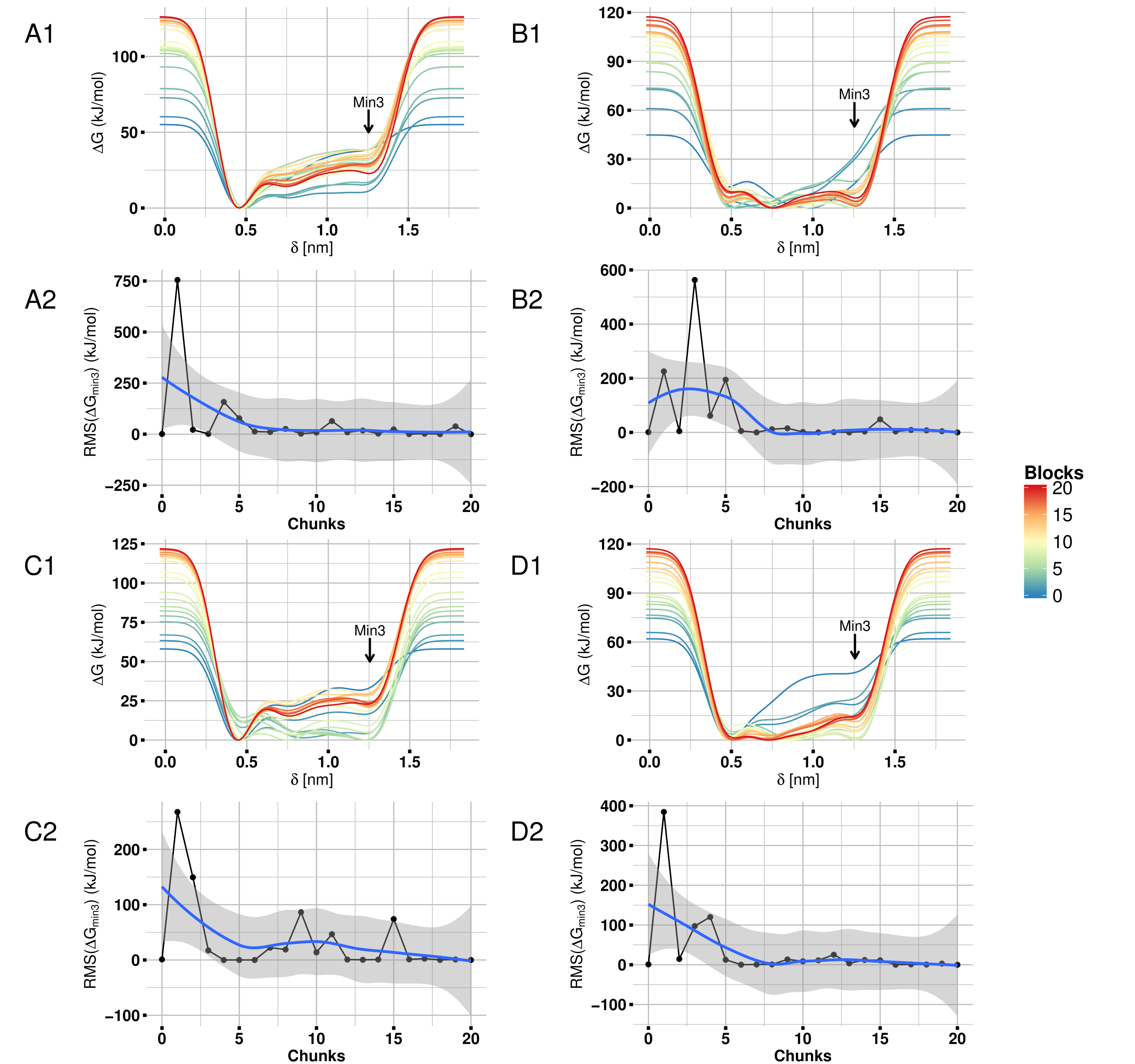

Supplement: S3 Fig — Time dependent free energy profiles on δ computed on successive blocks ([0, i*10ns] intervals) are overlapped. Convergence is achieved when the last blocks overlap. The colorbar indicates the blocks progression. This analysis has been repeated for the monomer(c) (A1), dimer(cc) (B1), tetramer(cccc) (C1) and tetramer(cocc) (D1). Moreover, the free energy value at δmin3=1.25 nm have been obtained from the ith profile. Then, the root mean square of two consecutive profiles is computed. RMS(ΔGmin3(δ))=[ΔG(i)min3(δ)-ΔG(i-1)Min3(δ)]. Blue line is a local regression fit (loess [46]), gray shadows confidence intervals from loess fit. Monomer(c) (A2), dimer(cc) (B2), tetramer(cccc) (C2) and tetramer(cocc) (D2). (TIFF) [file pcbi.1005202.s008.tiff]

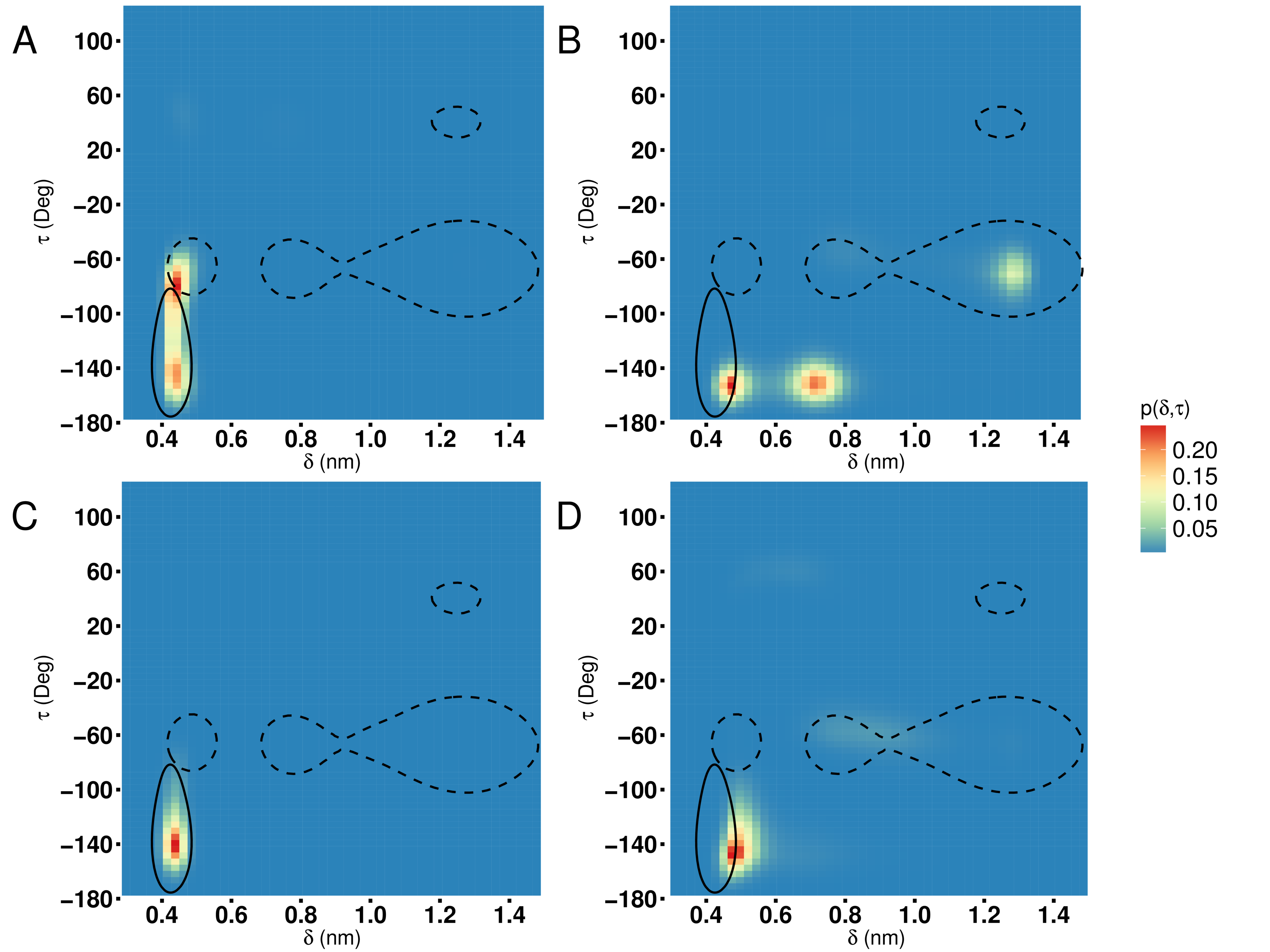

Supplement: S4 Fig — Joint probability density functions obtained from metadynamics simulations on the collective variables δ and τ for (A) Monomer(c), (B) Dimer(cc), (C) Tetramer(cccc), and (D) Tetramer(cocc). Superposed contour lines define regions which enclose 90% of the conformations sampled by the c (continuous) and o (dashed) form during 300 ns standard MD simulations. (TIFF) [file pcbi.1005202.s009.tiff]

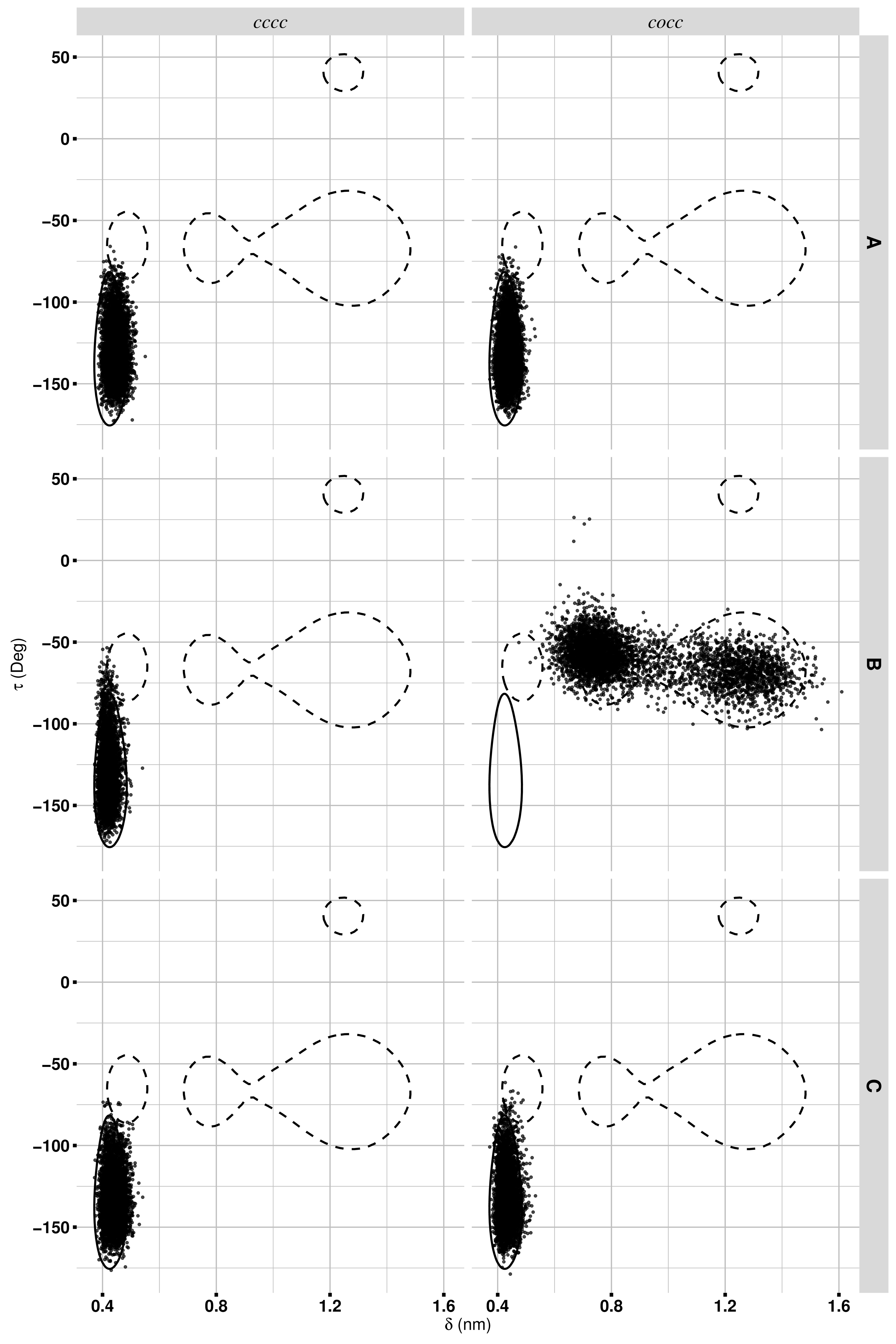

Supplement: S5 Fig — Values of δ and τ are shown for chains A, B, and C in tetramer(cccc) and tetramer(cocc) from MetaD simulations. Superposed contour lines define regions which enclose 90% of the conformations sampled by the c (continuous) and o (dashed) form during 300 ns standard MD simulations. As visible from the scatter plot, chains A, B, and C, which have not been subjected to the MetaD bias, remain in their initial conformation. (TIFF) [file pcbi.1005202.s010.tiff]

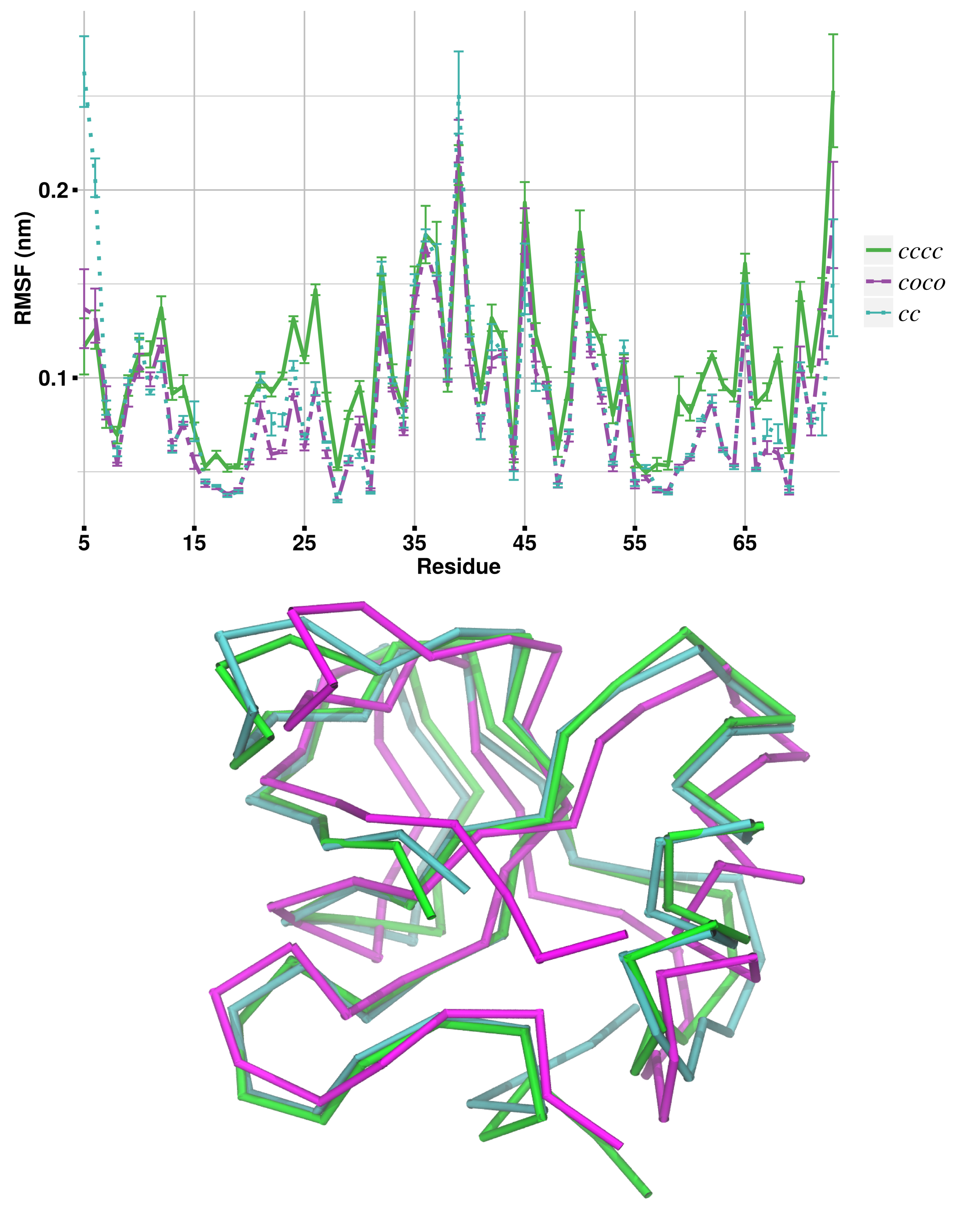

Supplement: S6 Fig — The root mean squared fluctuation (RMSF, i.e. the standard deviation) of atomic positions was computed for chain C from tetramer(cccc), tetramer(coco), and dimer(cc) from standard MD simulations. From the atomic RMSF the residue average is computed. Trajectories have been split into 5 blocks and the RMSF was computed on each block. On top, the RMSF profiles are shown as mean ± standard error of the mean, n = 5. The average difference between the three RMSF profiles expressed as root mean square is 0.07 nm. Bottom, the structures (last trajectory frame) of chain C in tetramer(cccc) (purple), tetramer(coco) (green) and dimer(cc) (cyan) have been superposed. The root mean square deviation (RMSD) between the atomic position of tetramer(cccc) or dimer(cc) and tetramer(coco) is less than 0.15 nm (hydrogens excluded). (TIFF) [file pcbi.1005202.s011.tiff]

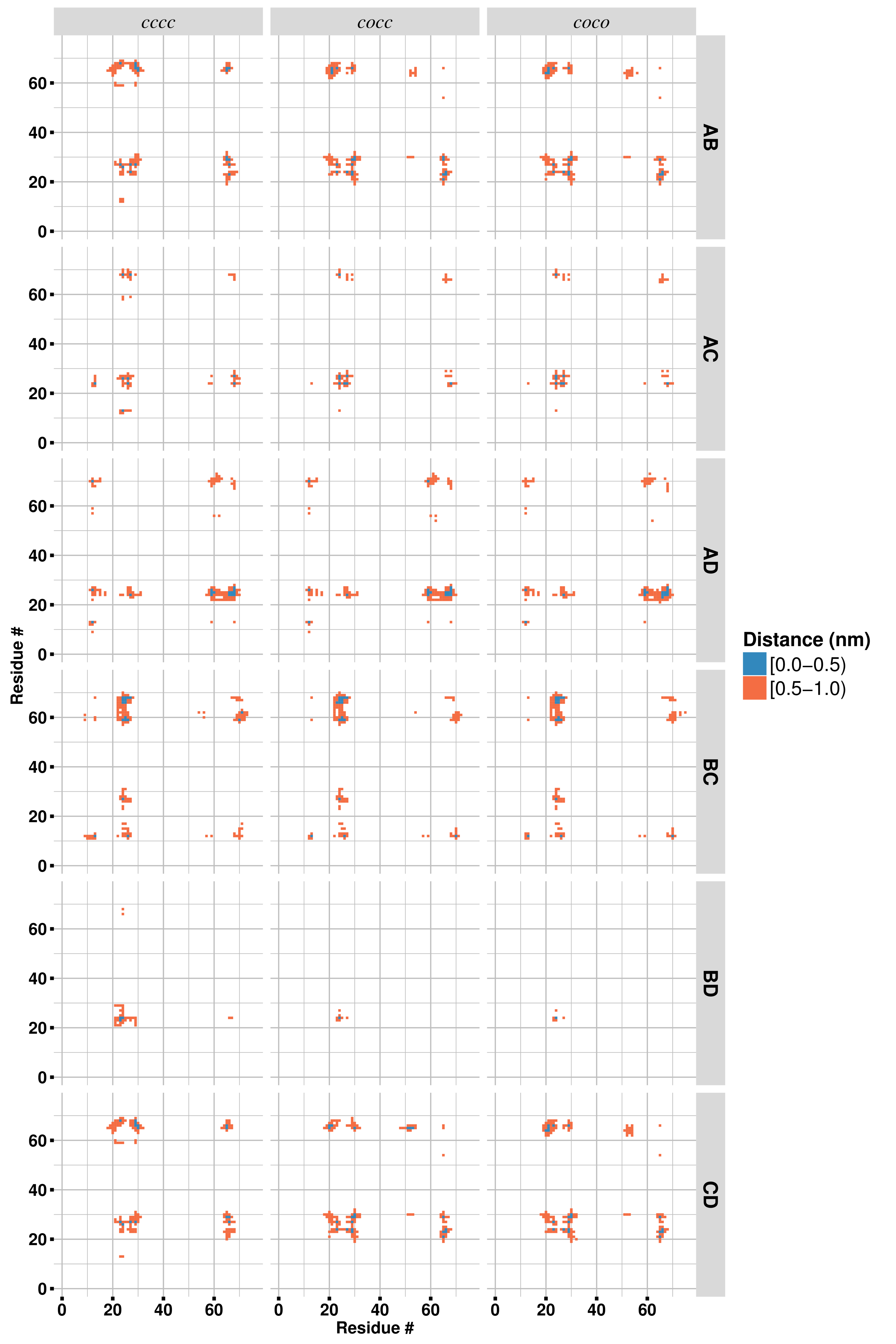

Supplement: S7 Fig — Residues distance maps computed for the chain pairs A/B, A/C, A/D, B/C, B/D, and C/D within tetramer(cccc), tetramer(cocc), and tetramer(coco). Distance maps have been obtained by measuring the smallest distances between residue pairs (heavy atoms only) for all trajectory frames and averaging over time. Analyses have been performed on standard MD simulations. See Method Section for further details. (TIFF) [file pcbi.1005202.s012.tiff]
